# Supplementary material for: Integration of GWAS and RNA-Seq Analysis to Identify SNPs and Candidate Genes Associated with Alkali Stress Tolerance at the Germination Stage in Mung Bean
Source: Genes (Basel). 2023 Jun 19;14(6):1294. doi: 10.3390/genes14061294 (PMC10298294; doi:10.3390/genes14061294)
Supplement: Supplementary file 1 [file genes-14-01294-s001.zip › Supplementary Materials/Table S1. List of mungbean accessions used in this study.pdf]

Table S1. List of mungbean accessions used in this study.

Code	Sequencing No.	Accession name	Province in China
1	Jl1	Gong lv 2	Jilin
2	Jl2	Gong lv 4	Jilin
3	Jl3	Ji lin lv 7	Jilin
4	Jl4	Ji lin lv 8	Jilin
5	Jl5	Ji lin lv 9	Jilin
6	Jl6	Ji lin lv 10	Jilin
7	Jl7	Tao lv 218	Jilin
8	Jl8	Tao lv 3	Jilin
9	Jl9	Tao lv 5	Jilin
10	Jl10	L4374	Jilin
11	Jl11	2088	Jilin
12	Hn1	He nan hei lv dou	Henan
13	Hb1	Bao 956-6	Hebei
14	Sd1	Wei 8901-32	Shandong
15	Bjjz1	Ming lv 1	Beijing
16	Img1	C0655	Inner Mongolia
17	Bjjz2	XLD2	Beijing
18	Bjjz3	XLD9	Beijing
19	Bjjz4	XLD8	Beijing
20	Hb2	Ji lv 2	Hebei
21	Bjjz5	XLD4	Beijing
22	Sd2	C1147	Shandong
23	Bj1	Zhong lv 2	Beijing
24	Sd3	C1013	Shandong
25	Bjjz6	BLS98051	Beijing
26	Bjjz7	XLD5	Beijing
27	Bjjz8	XLD3	Beijing
28	Bjjz9	XLD6	Beijing
29	Bjjz10	XLD1	Beijing
30	Hb3	Ji lv 7	Hebei
31	Img2	Qing shui he lv dou	Inner Mongolia
32	Sd4	Wei 9002-341	Shandong
33	Hb4	Bao 942-40-2	Hebei
34	Img3	C0595	Inner Mongolia
35	Bjjz11	BL93478-2	Beijing
36	Hb5	Ji lv 8	Hebei
37	Hub1	E lv 3	Hubei
38	Hub2	E lv 4	Hubei
39	Hlj1	Lv feng 2	Heilongjiang
40	Hlj2	Lv feng 5	Heilongjiang
41	Hb6	Zhang lv 3	Hebei
42	Gx1	Gui lv dou L74	Guangxi
43	Sx1	Jin lv dou 6	Shanxi
44	Sx2	Jin lv dou 7	Shanxi
45	Sx3	Jin lv dou 8	Shanxi
46	Sx4	Hei zhen zhu	Shanxi
47	Sd5	Wei lv 5	Shandong
48	Sd6	Wei lv 9	Shandong
49	Cq1	Yu hei lv 3	Chongqing
50	Cq2	Yu hei lv 4	Chongqing

51	Hb7	0816 mao-3	Hebei
52	Hb8	0506 fan hei-1-5-2-1	Hebei
53	Hb9	HN0904-2-1-1-1	Hebei
54	Hb10	0918-5-4-1-1	Hebei
55	Hb11	518	Hebei
56	Hb12	0616-4	Hebei
57	Js1	Su lv 3	Jiangsu
58	Js2	Su lv 4	Jiangsu
59	Js3	Su lv 5	Jiangsu
60	Js4	Su lv 6	Jiangsu
61	Js5	Su lv L13013	Jiangsu
62	Sx5	Da tong xiao ming lv dou	Shanxi
63	Sx6	Jin lv dou 9	Shanxi
64	Bj2	Zhong lv 4	Beijing
65	Bj3	Zhong lv 5	Beijing
66	Bj4	Zhong lv 6	Beijing
67	Bj5	Zhong lv 7	Beijing
68	Bj6	Zhong lv 9	Beijing
69	Bj7	Zhong lv 10	Beijing
70	Hb13	Bao 942	Hebei
71	Hb14	Zhang jia kou ying ge lv dou	Hebei
72	Sx7	Jin lv dou 3	Shanxi
73	Img4	Nei meng gu lv dou	Inner Mongolia
74	Sd7	Wei lv 7	Shandong
75	Sd8	Wei lv 8	Shandong
76	Ln1	Liao lv 8	Liaoning
77	Hlj3	Lv feng 3	Heilongjiang
78	Jl12	Ji lin 3	Jilin
79	Jl13	Bai lv 6	Jilin
80	Jl14	Bai lv 8	Jilin
81	Bj8	Zhong lv 11	Beijing
82	Hb15	Ji lv 9	Hebei
83	Bj9	Zhong lv 8	Beijing
84	Js6	Su lv 2	Jiangsu
85	Jl15	Ji lin lv 5	Jilin
86	Bj10	Zhong lv 14	Beijing
87	Img5	Ke lv 1	Inner Mongolia
88	Ln2	Liao lv 10	Liaoning
89	Hb16	Ji lv 11	Hebei
90	Jl16	Ji lin lv 6	Jilin
91	Bj11	Zhong lv 12	Beijing
92	Jl17	Bai lv 11	Jilin
93	Hb17	Ji lv 10	Hebei
94	Hlj4	Nen lv 2	Heilongjiang
95	Bj12	Pin lv 2011-06	Beijing
96	Bj13	Pin lv 2011-12	Beijing
97	Hb18	Ji lv 0816	Hebei
98	Hb19	Ji lv HN20810	Hebei
99	Js7	Su lv 16-10	Jiangsu
100	Js8	Su lv 15-11	Jiangsu
101	Hub3	E lv 5	Hubei
102	Sx8	1009-2-5	Shanxi

103	Jl18	Bai lv 10	Jilin
104	Sx9	Tong 1188326	Shanxi
105	Ln3	Liao lv 10L708-5	Liaoning
106	Hb20	Bao lv 200810-1	Hebei
107	Hb21	Bao lv 201012-7	Hebei
108	Img6	Ke lv 2	Inner Mongolia
109	Hlj5	142-139	Heilongjiang
110	Hlj6	122-225	Heilongjiang
111	Sd9	Wei lv 11	Shandong
112	Sd10	Wei lv 12	Shandong
113	HN2	Wan lv 2	Henan
114	Cq3	Yu lv 2	Chongqing
115	Sd11	Wei lv 05-8	Shandong
116	Hb22	Bao lv 200520	Hebei
117	Hb23	Ji lv 0204	Hebei
118	Hb24	Bao lv 200621	Hebei
119	Js9	Su lv 12-5	Jiangsu
120	Hb25	Bao lv 200644	Hebei
121	Hb26	Ji lv 0514	Hebei
122	Sx10	Jin 9908-34	Shanxi
123	Sd12	Wei lv 50934	Shandong
124	Sd13	Wei lv 52500	Shandong
125	Jl19	Ji lin lv 11	Jilin
126	Hb27	0802-4-2-1-2-1	Hebei
127	Hb28	HN1023-7-2	Hebei
128	Sx11	Tong 11411	Shanxi
129	Jl20	Bai lv 13	Jilin
130	Jl21	Da ying ge lv 935	Jilin
131	Ln4	Liao lv p13-02	Liaoning
132	Ln5	Liao lv 10L701	Liaoning
133	Hb29	Zhang lv 2	Hebei
134	Js10	Su lv 19-013	Jiangsu
135	Js11	Su lv 19-118	Jiangsu
136	HN3	Wan lv 5	Henan
137	HN4	Wan lv 6	Henan
138	Hlj7	132-346	Heilongjiang
139	Hlj8	112-285	Heilongjiang
140	Cq4	Yu lv 9	Chongqing
141	Bj14	Pin lv 2014-129	Beijing
142	Bj15	Pin lv 2014-124	Beijing
143	Hb30	Ji lv 13	Hebei
144	Bj16	LD036	Beijing
145	Bj17	LD041	Beijing
146	Bj18	LD042	Beijing
147	Bj19	LD091	Beijing
148	Tj1	Nong jia 13	Tianjin
149	Tj2	Nong jia 19	Tianjin
150	Bj20	LD031	Beijing
151	Hb31	Huang se ji dou	Hebei
152	Hb32	Tang shan lv dou	Hebei
153	Sd14	Yi du ming lv	Shandong
154	Hb33	Xiang he lv dou	Hebei

155	Sx12	Da yang lv dou	Shanxi
156	Hb34	Da huang lv dou	Hebei
157	Hb35	Qi xia ming lv	Hebei
158	Hb36	LD0135	Hebei
159	Hb37	Lu shi lv dou	Hebei
160	Hb38	Xiao li lv dou	Hebei
161	Jl22	Chang jia lv dou	Jilin
162	Sx13	Da lv dou	Shanxi
163	Sx14	Er bo zao	Shanxi
164	Sx15	Huang ge lv	Shanxi
165	Sx16	Da yang lv dou	Shanxi
166	Sx17	He nan hei lv dou	Shanxi
167	Img8	Tian shan ming lv	Inner Mongolia
168	Img7	Chi feng lv dou	Inner Mongolia
169	Jl23	Ju yuan lv dou	Jilin
170	Jl24	Xiao huang lv dou	Jilin
171	Jl25	Ji lv 9346	Jilin
172	Jl26	Yu shu da lv dou	Jilin
173	Jl27	Fei cui lv	Jilin
174	Ah1	Wan ke lv 1	Anhui
175	Ah2	Wan ke lv 2	Anhui
176	Ah3	Wan ke lv 3	Anhui
177	Ah4	Wan ke lv 4	Anhui
178	Ah5	Wan ke lv 5	Anhui
179	Ah6	Wan ke lv 6	Anhui
180	HN5	Yu lv 2	Henan
181	HN6	Yu lv 4	Henan
182	HN7	Zheng 08-103-04	Henan
183	HN8	Zhen 07-70	Henan
184	HN9	Zhen hei lv 1	Henan
185	HN10	Zheng lv 8	Henan
186	HN11	Zheng lv 11	Henan
187	HN12	Zheng lv 12	Henan
188	HN13	Zheng lv 17	Henan
189	HN14	Guo lv 3	Henan
190	HN15	Q yi lv 1	Henan
191	HN16	Q yi lv 2	Henan
192	HN17	Wan lv 1	Henan
193	HN18	An 07-3	Henan
194	HN19	An lv 8	Henan
195	HN20	An lv 10	Henan
196	HN21	An lv 11	Henan
197	Ah7	AHM1	Anhui
198	Ah8	AHM2	Anhui
199	Ah9	AHM3	Anhui
200	Ah10	AHM4	Anhui
201	Ah11	AHM5	Anhui
202	Ah12	AHM6	Anhui
203	Ah13	AHM7	Anhui
204	Ah14	AHM8	Anhui
205	Ah15	AHM9	Anhui
206	Ah16	AHM10	Anhui

207	Ah17	AHM11	Anhui
208	Ah18	AHM12	Anhui
209	Ah19	AHM13	Anhui
210	Ah20	AHM14	Anhui
211	Ah21	AHM15	Anhui
212	Ah22	AHM16	Anhui
213	Ah23	AHM17	Anhui
214	Ah24	AHM18	Anhui
215	Ah25	AHM19	Anhui
216	Ah26	AHM20	Anhui
217	HN22	Yu lv 1	Henan
218	HN23	Yu lv 3	Henan
219	HN24	Yu lv 5	Henan
220	HN25	Guo lv 1	Henan
221	HN26	Guo lv 2	Henan
222	HN27	An 9910	Henan
223	HN28	Zheng 03-94	Henan
224	HN29	Zheng lv 5	Henan
225	HN30	Ru yang lv dou	Henan
226	HN31	Wen xian lv dou	Henan
227	HN32	Deng xian lv dou	Henan
228	Tj3	Nong jia 3	Tianjin
229	Tj4	Nong jia 15	Tianjin
230	Tj5	Nong jia 25	Tianjin
231	Tj6	Nong jia 35	Tianjin
232	Bj21	LD035	Beijing
233	Bj22	LD043	Beijing
234	Bj23	LD045	Beijing
235	Bj24	LD051	Beijing
236	Jl28	Gong jiao 1099	Jilin
237	Jl29	1123	Jilin
238	Jl30	2154	Jilin
239	Jl31	3563	Jilin
240	Jl32	3564	Jilin
241	Jl33	9283	Jilin
242	Sx18	84-22	Shanxi
243	Jl34	r 68-27	Jilin
244	Jl35	Lv dou 103	Jilin
245	Jl36	Lv dou 109	Jilin
246	Jl37	Lv dou 711	Jilin
247	Jl38	Lv dou 716	Jilin
248	Jl39	Lv dou 740	Jilin
249	Jl40	Lv dou 822	Jilin
250	Jl41	Lv dou 827	Jilin
251	Jl42	Lv dou 833	Jilin
252	Jl43	Lv dou 423	Jilin
253	HN33	Zheng zhou 427	Henan
254	Ln6	Lv dou 12	Liaoning
255	Ln7	Lv dou 74	Liaoning
256	Ln8	Lv dou 82	Liaoning
257	Ln9	Lv dou 85	Liaoning
258	Ln10	Lv nong 8	Liaoning

259	Ln11	Lv nong 9	Liaoning
260	Hub4	E lv 1	Hubei
261	Js12	Xu yin 1	Jiangsu
262	Hb39	Ji yin 6	Hebei
263	Sx19	Jin yin 1	Shanxi
264	Sx20	Jin yin 2	Shanxi
265	Hlj9	Long lv 1	Heilongjiang
266	Hlj10	Lv feng 1	Heilongjiang
267	Sx21	Yin D76	Shanxi
268	Jl44	Lv dou (4)	Jilin
269	Jl45	Lv dou (7)	Jilin
270	Jl46	Lv dou 8	Jilin
271	Jl47	Lv dou 7	Jilin
272	Jl48	Tang lv	Jilin
273	Jl49	Da li chang jia lv	Jilin
274	Jl50	Lv dou wang	Jilin
275	Jl51	Ju jia lv dou	Jilin
276	Jl52	Lv duo shou	Jilin
277	Jl53	T 13	Jilin
