# Supplementary material for: Integration of GWAS and RNA-Seq Analysis to Identify SNPs and Candidate Genes Associated with Alkali Stress Tolerance at the Germination Stage in Mung Bean
Source: Genes (Basel). 2023 Jun 19;14(6):1294. doi: 10.3390/genes14061294 (PMC10298294; doi:10.3390/genes14061294)
Supplement: Supplementary file 1 [file genes-14-01294-s001.zip › Supplementary Materials/Table S2. The primer sequences used for qRT-PCR.pdf]

Table S2. The primer sequences used for qRT-PCR.

Gene	Forward primer (5'-3')	Reverse primer (5'-3')
jg5712	CCACGGCCTGCTTTTCAAAA	TGGACGTGCTGCAGGAATAA
jg6225	GTTTGGTGGTGGCACTGTTG	GGCAACCACCAAGTTTGTGT
jg7597	CCAAAGTTTCCGGTTTTCAA	ACCAATGGTTCAGGAAACGA
jg7600	CATCTTCAGCAGTGGGAGCA	AGAAGCAAGGGTGAAGGGTG
jg7622	TTGCGAGAACCTGGCGAATA	CAACACCGGAAATCGTCCCT
jg12912	TCATGGACTGTTGGAGGTGC	AAACTTTAGCCTGGGGTCCG
jg19402	CAAACTCGCCCTCTCTCTGG	TCTGTGGACCCTTGGATGAGA
jg19416	CAAAAAGGCTGCTTCTGGTC	AGGGAACACCAACAAATGGA
jg21301	GTGGTTCAGGAGGCAATGGA	GCACATTGGAAATCACGGGG
jg25807	CAATGCCAAACACGCCAAGA	GAACTCCAAGGCTTCGGTGA
jg25997	GCAGTGGACGAAGCTAACCT	TGCCAAAACGTAACCCTCGA
jg30941	CAGACCCATCAGTGCCCTAC	CATGGAAGGGTTGGTGGTGA
jg31682	GGAGGAGGAACATCAAAAGGGT	CAGTAGCTGGCTTCTCGTATCA
jg32966	ATTTGCCCTTACTGCGGTGA	GAACCAGACCTCCCAATCCG
VrActin	GGCATCCACGAGACAACA	AGCCTCCAATCCAGACAC
