# Supplementary material for: Integration of GWAS and RNA-Seq Analysis to Identify SNPs and Candidate Genes Associated with Alkali Stress Tolerance at the Germination Stage in Mung Bean
Source: Genes (Basel). 2023 Jun 19;14(6):1294. doi: 10.3390/genes14061294 (PMC10298294; doi:10.3390/genes14061294)
Supplement: Supplementary file 1 [file genes-14-01294-s001.zip › Supplementary Materials/Table S3. Phenotypic variation of GR and GI of the 277 mungbean accessions under control and alkali stress environments.pdf]

Table S3. Phenotypic variation of GR and GI of the 277 mungbean accessions under control and alkali stress environments.

		GR	GI
Control	Min	32.00%	3.39
	Median	98.70%	21.67
	Max	100.00%	25.00
	Mean	95.16%	20.74
	SD	0.08	3.61
	Kurtosis	-3.35	-1.43
	Skewness	16.16	2.48
	CV (%)	8.60	17.41
Alkali stress	Min	0.00%	0.00
	Median	30.70%	5.42
	Max	93.33%	19.33
	Mean	33.52%	6.15
	SD	0.23	4.31
	Skewness	0.45	0.70
	Kurtosis	-0.82	-0.33
	CV (%)	67.96	70.10

GR: Germination rate; GI: Germination index; Min: minimum; Max: maximum; *SD*: standard deviation; *CV*: coefficient of variation.
