# Supplementary material for: Integration of GWAS and RNA-Seq Analysis to Identify SNPs and Candidate Genes Associated with Alkali Stress Tolerance at the Germination Stage in Mung Bean
Source: Genes (Basel). 2023 Jun 19;14(6):1294. doi: 10.3390/genes14061294 (PMC10298294; doi:10.3390/genes14061294)
Supplement: Supplementary file 1 [file genes-14-01294-s001.zip › Supplementary Materials/Table S4. The 691 candidate genes related to alkali stress detected by GWAS.pdf]

Table S4. The 691 candidate genes related to alkali stress detected by GWAS.

Gene ID	Traits	Chr	Start	End	Pos	Function description
jg7594	RGR, RGI	3	1301765	1313414	1599285	NA
jg7595	RGR, RGI	3	1318449	1319222	1599285	NA
jg7596	RGR, RGI	3	1321253	1323070	1599285	Stem-specific protein TSJT1 OS=Nicotiana tabacum GN=TSJT1 PE=2 SV=1
jg7597	RGR, RGI	3	1348370	1348943	1599285	NA
jg7598	RGR, RGI	3	1356743	1356979	1599285	NA
jg7599	RGR, RGI	3	1456193	1457060	1599285	NA
jg7600	RGR, RGI	3	1458448	1461708	1599285	CBS domain-containing protein CBSX5 OS=Arabidopsis thaliana GN=CBSX5 PE=2 SV=2
jg7601	RGR, RGI	3	1462262	1469980	1599285	Ultraviolet-B receptor UVR8 OS=Arabidopsis thaliana GN=UVR8 PE=1 SV=1/
jg7602	RGR, RGI	3	1485635	1975048	1599285	NA
jg7603	RGR, RGI	3	1501168	1501500	1599285	NA
jg7604	RGR, RGI	3	1512615	1512938	1599285	NA
jg7605	RGR, RGI	3	1522130	1522852	1599285	NA
jg7606	RGR, RGI	3	1527892	1544297	1599285	Plant intracellular Ras-group-related LRR protein 6 OS=Oryza sativa subsp. japonica GN=IRL6 PE=2 SV=1
jg7607	RGR, RGI	3	1572669	1578689	1599285	Beta-glucosidase 18 OS=Oryza sativa subsp. japonica GN=BGLU18 PE=3 SV=2
jg7608	RGR, RGI	3	1596146	1596562	1599285	NA
jg7609	RGR, RGI	3	1617972	1624049	1599285	NA
jg7610	RGR, RGI	3	1656020	1659670	1599285	Probable inactive beta-glucosidase 14 OS=Oryza sativa subsp. japonica GN=BGLU14 PE=2 SV=2
jg7611	RGR, RGI	3	1679892	1685244	1599285	Nuclear/nucleolar GTPase 2 OS=Oryza sativa subsp. indica GN=NUG2 PE=3 SV=1
jg7612	RGR, RGI	3	1696311	1697082	1599285	NA
jg7613	RGR, RGI	3	1709781	1716099	1599285	MLO-like protein 11 OS=Arabidopsis thaliana GN=MLO11 PE=2 SV=1
jg7614	RGR, RGI	3	1717130	1727467	1599285	Choline transporter protein 1 OS=Arabidopsis thaliana GN=CHER1 PE=1 SV=1
jg7615	RGR, RGI	3	1743009	1753274	1599285	NA
jg7616	RGR, RGI	3	1761394	1761751	1599285	NA
jg7617	RGR, RGI	3	1805491	1806500	1599285	NA
jg7618	RGR, RGI	3	1822393	1829634	1599285	NA

jg7619	RGR, RGI	3	1833542	1838658	1599285	NA
jg7620	RGR, RGI	3	1842854	1844739	1599285	NA
jg7621	RGR, RGI	3	1844977	1845806	1599285	NA
jg7622	RGR, RGI	3	1850269	1850998	1599285	Defensin-like protein OS=Vigna unguiculata PE=3 SV=1
jg7623	RGR, RGI	3	1863674	1864085	1599285	NA
jg7624	RGR, RGI	3	1869149	1869573	1599285	NA
jg7625	RGR, RGI	3	1883405	1887218	1599285	Thioredoxin M4, chloroplastic OS=Arabidopsis thaliana GN=At3g15360 PE=2 SV=2
jg19402	RGR, RGI	6	39291070	39292427	39567121	Protein EXORDIUM OS=Arabidopsis thaliana GN=EXO PE=2 SV=1
jg19403	RGR, RGI	6	39313388	39313782	39567121	NA
jg19404	RGR, RGI	6	39330048	39331930	39567121	Protein EXORDIUM OS=Arabidopsis thaliana GN=EXO PE=2 SV=1
jg19405	RGR, RGI	6	39354947	39356216	39567121	Protein EXORDIUM OS=Arabidopsis thaliana GN=EXO PE=2 SV=1
jg19406	RGR, RGI	6	39362816	39363058	39567121	NA
jg19407	RGR, RGI	6	39381363	39388640	39567121	NA
jg19408	RGR, RGI	6	39408804	39411595	39567121	NA
jg19409	RGR, RGI	6	39424909	39425247	39567121	NA
jg19410	RGR, RGI	6	39428334	39428576	39567121	NA
jg19411	RGR, RGI	6	39429060	39431136	39567121	NA
jg19412	RGR, RGI	6	39435353	39435664	39567121	NA
jg19413	RGR, RGI	6	39448344	39448559	39567121	NA
jg19414	RGR, RGI	6	39451650	39453348	39567121	NA
jg19415	RGR, RGI	6	39460303	39461420	39567121	NA
jg19416	RGR, RGI	6	39462440	39466668	39567121	7-deoxyloganetin glucosyltransferase OS=Gardenia jasmiNAides GN=UGT85A24 PE=1 SV=1
jg19417	RGR, RGI	6	39467031	39468566	39567121	NA
jg19418	RGR, RGI	6	39504014	39504424	39567121	NA
jg19419	RGR, RGI	6	39508240	39515411	39567121	NA
jg19420	RGR, RGI	6	39516209	39516619	39567121	NA
jg19421	RGR, RGI	6	39520455	39521433	39567121	NA
jg19422	RGR, RGI	6	39539534	39543348	39567121	NA
jg19423	RGR, RGI	6	39571220	39573535	39567121	7-deoxyloganetin glucosyltransferase OS=Gardenia jasmiNAides GN=UGT85A24 PE=1 SV=1
jg19424	RGR, RGI	6	39579151	39580351	39567121	NA
jg19425	RGR, RGI	6	39586012	39588720	39567121	NA

jg19426	RGR, RGI	6	39584978	39598683	39567121	NA
jg19427	RGR, RGI	6	39604956	39605654	39567121	NA
jg19428	RGR, RGI	6	39630335	39630903	39567121	NA
jg19429	RGR, RGI	6	39631206	39633471	39567121	NA
jg19430	RGR, RGI	6	39678304	39684577	39567121	NA
jg19431	RGR, RGI	6	39691238	39693320	39567121	Putative RING-H2 finger protein ATL21B OS=Arabidopsis thaliana GN=ATL21B PE=3 SV=1
jg19432	RGR, RGI	6	39707759	39712965	39567121	NA
jg19433	RGR, RGI	6	39719124	39719493	39567121	NA
jg19434	RGR, RGI	6	39745124	39747783	39567121	NA
jg19435	RGR, RGI	6	39752014	39753008	39567121	NA
jg19436	RGR, RGI	6	39767668	39768318	39567121	NA
jg19437	RGR, RGI	6	39779253	39785619	39567121	NA
jg19438	RGR, RGI	6	39783803	39791511	39567121	Putative RING-H2 finger protein ATL21B OS=Arabidopsis thaliana GN=ATL21B PE=3 SV=1
jg19439	RGR, RGI	6	39790045	39791511	39567121	NA
jg19440	RGR, RGI	6	39793762	39796938	39567121	NA
jg19441	RGR, RGI	6	39787253	39833171	39567121	Rust resistance kinase Lr10 OS=Triticum aestivum GN=LRK10 PE=2 SV=1
jg19442	RGR, RGI	6	39855390	39857574	39567121	7-deoxyloganetin glucosyltransferase OS=Gardenia jasmiNAides GN=UGT85A24 PE=1 SV=1
jg19443	RGR, RGI	6	39860792	39862271	39567121	Protein TRANSPARENT TESTA 1 OS=Arabidopsis thaliana GN=TT1 PE=1 SV=1
jg25733	RGR	7	71976300	71979834	72278080	Protein PLASTID MOVEMENT IMPAIRED 1 OS=Arabidopsis thaliana GN=PMI1 PE=1 SV=1
jg25734	RGR	7	71980057	71983188	72278080	NA
jg25736	RGR	7	71994188	71998417	72278080	Sialyltransferase-like protein 2 OS=Arabidopsis thaliana GN=SIA2 PE=2 SV=1
jg25737	RGR	7	72000810	72004642	72278080	Pre-mRNA-splicing factor SPF27 homolog OS=Arabidopsis thaliana GN=MOS4 PE=1 SV=1
jg25738	RGR	7	72005350	72009853	72278080	Peroxisome biogenesis protein 3-2 OS=Arabidopsis thaliana GN=PEX3-2 PE=2 SV=1
jg25739	RGR	7	72011653	72013853	72278080	Guanine nucleotide-binding protein subunit beta-like protein OS=Glycine max PE=2 SV=1

jg25740	RGR	7	72017357	72022654	72278080	Actin-interacting protein 1-2 OS=Arabidopsis thaliana GN=AIP1-2 PE=2 SV=1
jg25741	RGR	7	72026199	72029075	72278080	Two-component response regulator ARR14 OS=Arabidopsis thaliana GN=ARR14 PE=1 SV=2
jg25742	RGR	7	72032781	72034933	72278080	Protein MOTHER of FT and TFL1 OS=Arabidopsis thaliana GN=MFT PE=1 SV=1
jg25743	RGR	7	72049946	72051983	72278080	NA
jg25744	RGR	7	72066693	72069412	72278080	Uncharacterized GPI-anchored protein At4g28100 OS=Arabidopsis thaliana GN=At4g28100 PE=1 SV=1
jg25745	RGR	7	72071232	72072585	72278080	Uncharacterized protein At5g39865 OS=Arabidopsis thaliana GN=At5g39865 PE=2 SV=1
jg25746	RGR	7	72073833	72076533	72278080	Pentatricopeptide repeat-containing protein At4g02750 OS=Arabidopsis thaliana GN=PCMP-H24 PE=3 SV=1
jg25747	RGR	7	72076796	72080382	72278080	Pentatricopeptide repeat-containing protein At4g02750 OS=Arabidopsis thaliana GN=PCMP-H24 PE=3 SV=1
jg25748	RGR	7	72080681	72085787	72278080	Mitogen-activated protein kinase 9 OS=Arabidopsis thaliana GN=MPK9 PE=2 SV=2
jg25749	RGR	7	72089669	72093250	72278080	HMG-Y-related protein A OS=Zea mays GN=HMGIY2 PE=1 SV=1
jg25750	RGR	7	72094036	72096557	72278080	Probable arabiNAsyltransferase ARAD1 OS=Arabidopsis thaliana GN=ARAD1 PE=1 SV=1
jg25751	RGR	7	72101461	72103266	72278080	Probable serine/threonine-protein kinase SIS8 OS=Arabidopsis thaliana GN=SIS8 PE=1 SV=1
jg25752	RGR	7	72101461	72110182	72278080	Probable serine/threonine-protein kinase SIS8 OS=Arabidopsis thaliana GN=SIS8 PE=1 SV=1
jg25753	RGR	7	72111096	72112777	72278080	Peptidyl-prolyl cis-trans isomerase FKBP17-2, chloroplastic OS=Arabidopsis thaliana GN=FKBP17-2 PE=1 SV=1
jg25754	RGR	7	72114861	72119342	72278080	Eukaryotic translation initiation factor 3 subunit C OS=Medicago truncatula GN=TIF3C1 PE=2 SV=1
jg25755	RGR	7	72121144	72125288	72278080	NA
jg25756	RGR	7	72131995	72133983	72278080	NA
jg25757	RGR	7	72134981	72136033	72278080	NA
jg25758	RGR	7	72144568	72149876	72278080	Pentatricopeptide repeat-containing protein At2g01390 OS=Arabidopsis thaliana GN=At2g01390/At2g01380 PE=2 SV=2
jg25759	RGR	7	72150360	72156723	72278080	Golgin candidate 2 OS=Arabidopsis thaliana GN=GC2 PE=1 SV=1

jg25760	RGR	7	72157448	72159781	72278080	Ras-related protein RABA6a OS=Arabidopsis thaliana GN=RABA6A PE=2 SV=1
jg25761	RGR	7	72162408	72163866	72278080	Calcium-binding allergen Ole e 8 OS=Olea europaea PE=1 SV=1
jg25762	RGR	7	72166533	72168617	72278080	Thaumatin-like protein OS=Arabidopsis thaliana GN=At1g18250 PE=2 SV=2
jg25763	RGR	7	72172851	72176026	72278080	WUSCHEL-related homeobox 1 OS=Arabidopsis thaliana GN=WOX1 PE=2 SV=2
jg25764	RGR	7	72179790	72182717	72278080	Pentatricopeptide repeat-containing protein At2g01390 OS=Arabidopsis thaliana GN=At2g01390/At2g01380 PE=2 SV=2
jg25765	RGR	7	72183687	72186053	72278080	Tubulin beta-6 chain OS=Zea mays GN=TUBB6 PE=2 SV=1
jg25766	RGR	7	72186262	72189852	72278080	ADP,ATP carrier protein ER-ANT1 OS=Arabidopsis thaliana GN=ER-ANT1 PE=2 SV=2
jg25767	RGR	7	72191589	72197849	72278080	Translocon at the outer membrane of chloroplasts 64 OS=Pisum sativum GN=TOC64 PE=1 SV=1
jg25768	RGR	7	72198621	72202291	72278080	Protein C2-DOMAIN ABA-RELATED 4 OS=Arabidopsis thaliana GN=CAR4 PE=1 SV=1
jg25769	RGR	7	72199602	72202291	72278080	Protein C2-DOMAIN ABA-RELATED 4 OS=Arabidopsis thaliana GN=CAR4 PE=1 SV=1
jg25770	RGR	7	72203600	72206811	72278080	Auxin efflux carrier component 1a OS=Oryza sativa subsp. japonica GN=PIN1A PE=2 SV=1
jg25771	RGR	7	72209956	72214603	72278080	PhosphoethaNAlamine N-methyltransferase 1 OS=Arabidopsis thaliana GN=NMT1 PE=1 SV=1
jg25772	RGR	7	72215525	72218763	72278080	Protection of telomeres protein 1b OS=Arabidopsis thaliana GN=POT1B PE=1 SV=1
jg25773	RGR	7	72216575	72218763	72278080	Protection of telomeres protein 1a OS=Arabidopsis thaliana GN=POT1A PE=1 SV=1
jg25774	RGR	7	72222522	72224501	72278080	NA
jg25775	RGR	7	72224530	72225795	72278080	Protection of telomeres protein 1b OS=Arabidopsis thaliana GN=POT1B PE=1 SV=1
jg25776	RGR	7	72226710	72231811	72278080	Protection of telomeres protein 1b OS=Arabidopsis thaliana GN=POT1B PE=1 SV=1
jg25777	RGR	7	72232326	72237324	72278080	ERAD-associated E3 ubiquitin-protein ligase component HRD3A OS=Arabidopsis thaliana GN=HRD3A PE=1 SV=1

jg25778	RGR	7	72238290	72240035	72278080	ABC transporter I family member 17 OS=Arabidopsis thaliana GN=ABCI17 PE=2 SV=1
jg25779	RGR	7	72240038	72242178	72278080	Uncharacterized protein At3g17950 OS=Arabidopsis thaliana GN=Y-3 PE=1 SV=1
jg25780	RGR	7	72244960	72246935	72278080	NA
jg25781	RGR	7	72244960	72250452	72278080	NA
jg25783	RGR	7	72264814	72269671	72278080	ABC transporter G family member 2 OS=Arabidopsis thaliana GN=ABCG2 PE=2 SV=1
jg25784	RGR	7	72266409	72273879	72278080	TPR repeat-containing thioredoxin TDX OS=Arabidopsis thaliana GN=TDX PE=1 SV=1
jg25785	RGR	7	72276786	72279233	72278080	Factor of DNA methylation 1 OS=Arabidopsis thaliana GN=FDM1 PE=1 SV=1
jg25786	RGR	7	72282086	72285488	72278080	Probable inactive receptor kinase At1g48480 OS=Arabidopsis thaliana GN=RKL1 PE=2 SV=1
jg25787	RGR	7	72288913	72293093	72278080	NF-X1-type zinc finger protein NFXL1 OS=Arabidopsis thaliana GN=NFXL1 PE=1 SV=1
jg25788	RGR	7	72296867	72301102	72278080	Potassium transporter 25 OS=Oryza sativa subsp. japonica GN=HAK25 PE=2 SV=1
jg25789	RGR	7	72302896	72320681	72278080	Potassium channel SKOR OS=Arabidopsis thaliana GN=SKOR PE=1 SV=1
jg25790	RGR	7	72302896	72314364	72278080	Retrovirus-related Pol polyprotein from transposon TNT 1-94 OS=Nicotiana tabacum PE=2 SV=1
jg25791	RGR	7	72317987	72320681	72278080	UV-B-induced protein At3g17800, chloroplastic OS=Arabidopsis thaliana GN=At3g17800 PE=2 SV=1
jg25792	RGR	7	72322573	72324808	72278080	Purple acid phosphatase 3 OS=Arabidopsis thaliana GN=PAP3 PE=2 SV=1
jg25793	RGR	7	72326217	72328418	72278080	Purple acid phosphatase 17 OS=Arabidopsis thaliana GN=PAP17 PE=2 SV=1
jg25794	RGR	7	72330095	72333646	72278080	Annexin D5 OS=Arabidopsis thaliana GN=ANN5 PE=2 SV=2
jg25796	RGR	7	72341700	72344112	72278080	Zinc-finger homeodomain protein 2 OS=Arabidopsis thaliana GN=ZHD1 PE=1 SV=1
jg25797	RGR	7	72355462	72358358	72278080	Protein SHOOT GRAVITROPISM 5 OS=Arabidopsis thaliana GN=SGR5 PE=1 SV=1
jg25798	RGR	7	72362149	72366590	72278080	NA

jg25801	RGR	7	72382175	72388579	72278080	Putative D-cysteine desulfhydrase 1, mitochondrial OS=Oryza sativa subsp. japonica GN=Os02g0773300 PE=2 SV=2
jg25802	RGR	7	72388725	72394277	72278080	NA
jg25803	RGR	7	72400092	72403648	72278080	Homeobox-leucine zipper protein HDG11 OS=Arabidopsis thaliana GN=HDG11 PE=1 SV=1
jg25804	RGR	7	72408706	72409602	72278080	Universal stress protein A-like protein OS=Arabidopsis thaliana GN=At3g01520 PE=1 SV=2
jg25805	RGR	7	72414575	72417060	72278080	NA
jg25806	RGR	7	72418532	72424378	72278080	Rhodanese-like domain-containing protein 8, chloroplastic OS=Arabidopsis thaliana GN=STR8 PE=4 SV=1
jg25807	RGR	7	72425571	72429111	72278080	Probable ADP-ribosylation factor GTPase-activating protein AGD15 OS=Arabidopsis thaliana GN=AGD15 PE=2 SV=1
jg25808	RGR	7	72434170	72434478	72278080	NA
jg25809	RGR	7	72435410	72439085	72278080	NA
jg25810	RGR	7	72435410	72445981	72278080	Kinesin-like protein KIN-13A OS=Arabidopsis thaliana GN=KIN13A PE=1 SV=1
jg25811	RGR	7	72442411	72445981	72278080	Kinesin-like protein KIN-13A OS=Arabidopsis thaliana GN=KIN13A PE=1 SV=1
jg25812	RGR	7	72442411	72445981	72278080	Kinesin-like protein KIN-13A OS=Arabidopsis thaliana GN=KIN13A PE=1 SV=1
jg25813	RGR	7	72449149	72459437	72278080	Glucose-6-phosphate 1-dehydrogenase 2, chloroplastic OS=Arabidopsis thaliana GN=At5g13110 PE=1 SV=2
jg25814	RGR	7	72449149	72461204	72278080	Glucose-6-phosphate 1-dehydrogenase 3, chloroplastic OS=Arabidopsis thaliana GN=At1g24280 PE=1 SV=2
jg25815	RGR	7	72483442	72486923	72278080	Two-component response regulator ARR14 OS=Arabidopsis thaliana GN=ARR14 PE=1 SV=2
jg34109	RGR	11	7340081	7350415	7640538	DNA mismatch repair protein MLH3 OS=Arabidopsis thaliana GN=MLH3 PE=2 SV=2
jg34110	RGR	11	7358792	7362733	7640538	NA
jg34111	RGR	11	7360398	7362733	7640538	NA
jg34113	RGR	11	7371932	7374185	7640538	AmiNA acid transporter AVT3C OS=Arabidopsis thaliana GN=AVT3C PE=1 SV=1
jg34114	RGR	11	7382569	7386810	7640538	Pentatricopeptide repeat-containing protein At2g17525, mitochondrial OS=Arabidopsis thaliana GN=At2g17525 PE=2 SV=2

jg34115	RGR	11	7384316	7386810	7640538	Pentatricopeptide repeat-containing protein At2g17525, mitochondrial OS=Arabidopsis thaliana GN=At2g17525 PE=2 SV=2
jg34116	RGR	11	7387436	7390438	7640538	Probable protein phosphatase 2C 47 OS=Arabidopsis thaliana GN=At3g51470 PE=1 SV=1
jg34117	RGR	11	7391841	7396845	7640538	Glutamate receptor 3.6 OS=Arabidopsis thaliana GN=GLR3.6 PE=2 SV=1
jg34118	RGR	11	7397104	7403937	7640538	Serine/threonine-protein kinase/endoribonuclease IRE1a OS=Arabidopsis thaliana GN=IRE1A PE=1 SV=1
jg34119	RGR	11	7408755	7408964	7640538	NA
jg34120	RGR	11	7410274	7415991	7640538	Protein PIN-LIKES 7 OS=Arabidopsis thaliana GN=PILS7 PE=2 SV=1
jg34121	RGR	11	7420804	7424737	7640538	NA
jg34122	RGR	11	7431296	7435778	7640538	MLO-like protein 8 OS=Arabidopsis thaliana GN=MLO8 PE=1 SV=2
jg34123	RGR	11	7447911	7452317	7640538	RING-H2 finger protein ATL8 OS=Arabidopsis thaliana GN=ATL8 PE=2 SV=2
jg34124	RGR	11	7450955	7452317	7640538	RING-H2 finger protein ATL8 OS=Arabidopsis thaliana GN=ATL8 PE=2 SV=2
jg34125	RGR	11	7463200	7467066	7640538	MoNAsaccharide-sensing protein 3 OS=Arabidopsis thaliana GN=MSSP3 PE=2 SV=1
jg34126	RGR	11	7481644	7485756	7640538	Mitochondrial phosphate carrier protein 1, mitochondrial OS=Arabidopsis thaliana GN=MPT1 PE=2 SV=1
jg34127	RGR	11	7490363	7493668	7640538	Plant intracellular Ras-group-related LRR protein 4 OS=Arabidopsis thaliana GN=PIRL4 PE=1 SV=1
jg34129	RGR	11	7502644	7503773	7640538	NA
jg34133	RGR	11	7593990	7594217	7640538	NA
jg34134	RGR	11	7598669	7601569	7640538	NA
jg34135	RGR	11	7602469	7607412	7640538	Thioredoxin reductase 2 OS=Arabidopsis thaliana GN=NTR2 PE=2 SV=2
jg34136	RGR	11	7612102	7612881	7640538	NA
jg34137	RGR	11	7617130	7624134	7640538	AT-rich interactive domain-containing protein 3 OS=Arabidopsis thaliana GN=ARID3 PE=1 SV=1
jg34138	RGR	11	7635158	7639715	7640538	Protein PIN-LIKES 3 OS=Arabidopsis thaliana GN=PILS3 PE=2 SV=1
jg34139	RGR	11	7640083	7640609	7640538	NA
jg34140	RGR	11	7643383	7648715	7640538	NA

jg34141	RGR	11	7648983	7653262	7640538	Protein FAR1-RELATED SEQUENCE 5 OS=Arabidopsis thaliana GN=FRS5 PE=1 SV=1
jg34142	RGR	11	7654885	7662765	7640538	Diacylglycerol O-acyltransferase 2D OS=Glycine max GN=DGAT2D PE=1 SV=1
jg34144	RGR	11	7680708	7682416	7640538	Succinate dehydrogenase [ubiquiNAne] iron-sulfur subunit 2, mitochondrial OS=Arabidopsis thaliana GN=SDH2-2 PE=1 SV=2
jg34146	RGR	11	7695144	7695539	7640538	NA
jg34147	RGR	11	7712814	7717470	7640538	Subtilisin-like protease SBT5.3 OS=Arabidopsis thaliana GN=AIR3 PE=2 SV=1
jg34148	RGR	11	7717929	7719251	7640538	NA
jg34149	RGR	11	7725450	7730454	7640538	Probable L-type lectin-domain containing receptor kinase S.5 OS=Arabidopsis thaliana GN=LECRKS5 PE=2 SV=1
jg34150	RGR	11	7727339	7730454	7640538	2-oxoglutarate-dependent dioxygenase DAO OS=Oryza sativa subsp. japonica GN=DAO PE=2 SV=2
jg34151	RGR	11	7731640	7733211	7640538	2-oxoglutarate-dependent dioxygenase DAO OS=Oryza sativa subsp. japonica GN=DAO PE=2 SV=2
jg34152	RGR	11	7737471	7739127	7640538	2-oxoglutarate-dependent dioxygenase DAO OS=Oryza sativa subsp. japonica GN=DAO PE=2 SV=2
jg34153	RGR	11	7744267	7745740	7640538	2-oxoglutarate-dependent dioxygenase DAO OS=Oryza sativa subsp. japonica GN=DAO PE=2 SV=2
jg34154	RGR	11	7751466	7760617	7640538	Kinesin-like protein KIN-14E OS=Arabidopsis thaliana GN=KIN14E PE=1 SV=1
jg34155	RGR	11	7772806	7780563	7640538	Protein root UVB sensitive 1, chloroplastic OS=Arabidopsis thaliana GN=RUS1 PE=1 SV=1
jg34157	RGR	11	7772806	7780563	7640538	Protein root UVB sensitive 1, chloroplastic OS=Arabidopsis thaliana GN=RUS1 PE=1 SV=1
jg34158	RGR	11	7781871	7783517	7640538	Pentatricopeptide repeat-containing protein At1g31790 OS=Arabidopsis thaliana GN=PCMP-A1 PE=2 SV=1
jg34159	RGR	11	7784579	7787082	7640538	Probable caroteNAid cleavage dioxygenase 4, chloroplastic OS=Arabidopsis thaliana GN=CCD4 PE=1 SV=1
jg34160	RGR	11	7791183	7794908	7640538	Probable apyrase 7 OS=Arabidopsis thaliana GN=APY7 PE=2 SV=1
jg34161	RGR	11	7798007	7802655	7640538	WAT1-related protein At4g19185 OS=Arabidopsis thaliana GN=At4g19185 PE=2 SV=1

jg34162	RGR	11	7804249	7808230	7640538	WAT1-related protein At4g19185 OS=Arabidopsis thaliana GN=At4g19185 PE=2 SV=1
jg34163	RGR	11	7813181	7819295	7640538	NA
jg34165	RGR	11	7842496	7845728	7640538	ABC transporter G family member 14 OS=Arabidopsis thaliana GN=ABCG14 PE=2 SV=1
jg34166	RGR	11	7851127	7855908	7640538	Uncharacterized zinc finger CCHC domain-containing protein At4g19190 OS=Arabidopsis thaliana GN=At4g19190 PE=2 SV=1
jg34168	RGR	11	7871566	7872816	7640538	NA
jg34169	RGR	11	7874533	7877922	7640538	F-box protein SKIP31 OS=Arabidopsis thaliana GN=SKIP31 PE=1 SV=1
jg34170	RGR	11	7880744	7882974	7640538	NA
jg34171	RGR	11	7885391	7890742	7640538	ABC transporter E family member 2 OS=Arabidopsis thaliana GN=ABCE2 PE=2 SV=1
jg34172	RGR	11	7891703	7894837	7640538	Pentatricopeptide repeat-containing protein At4g19220, mitochondrial OS=Arabidopsis thaliana GN=PCMP-E2 PE=3 SV=2
jg34173	RGR	11	7894945	7903169	7640538	ArabiNAsyltransferase XEG113 OS=Arabidopsis thaliana GN=XEG113 PE=2 SV=1
jg34174	RGR	11	7935438	7938257	7640538	Abscisic acid 8'-hydroxylase 1 OS=Arabidopsis thaliana GN=CYP707A1 PE=2 SV=1
jg14061	RGI	1	12395195	12398889	12682868	Protein trichome birefringence-like 19 OS=Arabidopsis thaliana GN=TBL19 PE=3 SV=1
jg14062	RGI	1	12405490	12408171	12682868	NA
jg14063	RGI	1	12417053	12422691	12682868	BRCT domain-containing protein At4g02110 OS=Arabidopsis thaliana GN=At4g02110 PE=4 SV=3
jg14064	RGI	1	12425138	12436319	12682868	Cell division cycle protein 27 homolog B OS=Arabidopsis thaliana GN=CDC27B PE=1 SV=1
jg14066	RGI	1	12456206	12458604	12682868	NA
jg14067	RGI	1	12463841	12464059	12682868	NA
jg14070	RGI	1	12479636	12492993	12682868	NA
jg14071	RGI	1	12508981	12516054	12682868	Proline dehydrogenase 2, mitochondrial OS=Arabidopsis thaliana GN=POX2 PE=2 SV=1
jg14072	RGI	1	12515232	12518551	12682868	Proline dehydrogenase 2, mitochondrial OS=Arabidopsis thaliana GN=POX2 PE=2 SV=1
jg14073	RGI	1	12533544	12536105	12682868	NA

jg14074	RGI	1	12536492	12536725	12682868	NA
jg14075	RGI	1	12541538	12544085	12682868	Probable polygalacturonase OS=Vitis vinifera GN=GSVIVT00026920001 PE=1 SV=1
jg14076	RGI	1	12555180	12556077	12682868	NA
jg14077	RGI	1	12559986	12563889	12682868	Nuclear poly(A) polymerase 3 OS=Arabidopsis thaliana GN=PAPS3 PE=1 SV=1
jg14078	RGI	1	12565150	12570197	12682868	Remorin OS=Solanum tuberosum PE=1 SV=1
jg14079	RGI	1	12565150	12570197	12682868	Remorin OS=Solanum tuberosum PE=1 SV=1
jg14080	RGI	1	12577270	12579666	12682868	NA
jg14081	RGI	1	12581936	12583421	12682868	NA
jg14083	RGI	1	12612301	12616652	12682868	Protein CHROMOSOME TRANSMISSION FIDELITY 7 OS=Arabidopsis thaliana GN=CTF7 PE=1 SV=1
jg14084	RGI	1	12618579	12622723	12682868	Probable inactive ATP-dependent zinc metalloprotease FTSHI 3, chloroplastic OS=Arabidopsis thaliana GN=FTSHI3 PE=1 SV=1
jg14085	RGI	1	12624241	12627296	12682868	TMV resistance protein N OS=Nicotiana glutiNAsa GN=N PE=1 SV=1
jg14086	RGI	1	12633228	12637440	12682868	TMV resistance protein N OS=Nicotiana glutiNAsa GN=N PE=1 SV=1
jg14087	RGI	1	12649456	12649719	12682868	NA
jg14088	RGI	1	12674787	12678856	12682868	NA
jg14089	RGI	1	12680766	12683017	12682868	NA
jg14090	RGI	1	12718700	12723251	12682868	Protein NSP-INTERACTING KINASE 1 OS=Arabidopsis thaliana GN=NIK1 PE=1 SV=1
jg14091	RGI	1	12727824	12729930	12682868	60S ribosomal protein L9 OS=Pisum sativum GN=RPL9 PE=2 SV=1
jg14092	RGI	1	12739643	12741800	12682868	Zinc finger protein CONSTANS-LIKE 2 OS=Arabidopsis thaliana GN=COL2 PE=1 SV=1
jg14093	RGI	1	12764143	12768037	12682868	NA
jg14094	RGI	1	12787512	12790954	12682868	Calcium-transporting ATPase 12, plasma membrane-type OS=Arabidopsis thaliana GN=ACA12 PE=2 SV=1
jg14095	RGI	1	12788100	12790954	12682868	Calcium-transporting ATPase 12, plasma membrane-type OS=Arabidopsis thaliana GN=ACA12 PE=2 SV=1
jg14096	RGI	1	12792057	12792518	12682868	Probable prolyl 4-hydroxylase 7 OS=Arabidopsis thaliana GN=P4H7 PE=2 SV=1
jg14097	RGI	1	12797567	12801146	12682868	NA
jg14098	RGI	1	12805123	12807864	12682868	6-phosphogluconate dehydrogenase, decarboxylating 3 OS=Arabidopsis thaliana GN=At3g02360 PE=2 SV=1

jg14099	RGI	1	12811822	12814765	12682868	Transcription factor MYB83 OS=Arabidopsis thaliana GN=MYB83 PE=2 SV=1
jg14100	RGI	1	12825302	12828373	12682868	Probable galacturoNAsyltransferase 9 OS=Arabidopsis thaliana GN=GAUT9 PE=2 SV=1
jg14101	RGI	1	12836106	12838154	12682868	Basic leucine zipper 43 OS=Arabidopsis thaliana GN=BZIP43 PE=1 SV=1
jg14102	RGI	1	12859883	12861558	12682868	NA
jg14103	RGI	1	12883038	12884190	12682868	NA
jg14104	RGI	1	12884323	12884773	12682868	NA
jg14105	RGI	1	12890024	12890309	12682868	NA
jg14106	RGI	1	12890682	12892518	12682868	E3 ubiquitin-protein ligase RING1-like OS=Arabidopsis thaliana GN=At3g19950 PE=1 SV=1
jg14107	RGI	1	12895959	12900382	12682868	WPP domain-interacting tail-anchored protein 1 OS=Arabidopsis thaliana GN=WIT1 PE=1 SV=2
jg14108	RGI	1	12905019	12906422	12682868	NA
jg14109	RGI	1	12912961	12914956	12682868	NA
jg14110	RGI	1	12916615	12917436	12682868	Umecyanin OS=Armoracia rusticana PE=1 SV=1
jg14111	RGI	1	12922059	12926156	12682868	NA
jg14112	RGI	1	12927164	12927466	12682868	NA
jg14113	RGI	1	12928688	12933464	12682868	ATP sulfurylase 2 OS=Arabidopsis thaliana GN=APS2 PE=1 SV=1
jg14114	RGI	1	12935863	12936426	12682868	NA
jg14115	RGI	1	12946194	12948330	12682868	NA
jg14116	RGI	1	12960639	12961216	12682868	NA
jg14117	RGI	1	12968484	12972579	12682868	AmiNA acid transporter AVT6A OS=Arabidopsis thaliana GN=AVT6A PE=2 SV=1
jg14699	RGI	1	18983165	18991229	19279047	NA
jg14700	RGI	1	18999703	19012516	19279047	Succinate dehydrogenase [ubiquiNAne] iron-sulfur subunit 2, mitochondrial OS=Arabidopsis thaliana GN=SDH2-2 PE=1 SV=2
jg14701	RGI	1	18999703	19007333	19279047	Ent-kaur-16-ene synthase, chloroplastic OS=Cucurbita maxima PE=1 SV=1
jg14702	RGI	1	19010065	19012516	19279047	60S ribosomal protein L2, mitochondrial OS=Oryza sativa subsp. japonica GN=RPL2 PE=2 SV=2
jg14703	RGI	1	19010065	19012516	19279047	60S ribosomal protein L2, mitochondrial OS=Oryza sativa subsp. japonica GN=RPL2 PE=2 SV=2

jg14704	RGI	1	19050633	19054799	19279047	ALA-interacting subunit 3 OS=Arabidopsis thaliana GN=ALIS3 PE=1 SV=1
jg14705	RGI	1	19064845	19065237	19279047	NA
jg14706	RGI	1	19148948	19155299	19279047	Probable choline kinase 2 OS=Arabidopsis thaliana GN=At1g74320 PE=2 SV=1
jg14708	RGI	1	19189787	19200269	19279047	Type IV iNAsitol polyphosphate 5-phosphatase 3 OS=Arabidopsis thaliana GN=IP5P3 PE=1 SV=1
jg14709	RGI	1	19230646	19237541	19279047	30S ribosomal protein S1, chloroplastic OS=Arabidopsis thaliana GN=RPS1 PE=1 SV=1
jg14710	RGI	1	19258366	19260389	19279047	NA
jg14711	RGI	1	19340484	19341407	19279047	NA
jg14712	RGI	1	19357584	19365533	19279047	Protein unc-13 homolog OS=Arabidopsis thaliana GN=PATROL1 PE=2 SV=1
jg14713	RGI	1	19366189	19367438	19279047	NA
jg14714	RGI	1	19368652	19370647	19279047	Putative pentatricopeptide repeat-containing protein At5g59200, chloroplastic OS=Arabidopsis thaliana GN=PCMP-E41 PE=3 SV=1
jg14715	RGI	1	19383445	19385457	19279047	Putative pentatricopeptide repeat-containing protein At5g59200, chloroplastic OS=Arabidopsis thaliana GN=PCMP-E41 PE=3 SV=1
jg14716	RGI	1	19386654	19387903	19279047	NA
jg14717	RGI	1	19388587	19394608	19279047	Protein unc-13 homolog OS=Arabidopsis thaliana GN=PATROL1 PE=2 SV=1
jg14718	RGI	1	19424930	19425350	19279047	NA
jg14719	RGI	1	19445887	19448340	19279047	NA
jg10663	RGI	2	11664180	11666357	11936562	Peptide-N4-(N-acetyl-beta-glucosaminyl)asparagine amidase A OS=Prunus dulcis PE=1 SV=2
jg10664	RGI	2	11680552	11689084	11936562	ATP-dependent zinc metalloprotease FTSH 10, mitochondrial OS=Arabidopsis thaliana GN=FTSH10 PE=1 SV=1
jg10665	RGI	2	11698406	11702416	11936562	Phytosulfokine receptor 2 OS=Arabidopsis thaliana GN=PSKR2 PE=2 SV=1
jg10666	RGI	2	11703640	11704182	11936562	NA
jg10667	RGI	2	11706541	11707932	11936562	NA
jg10669	RGI	2	11729067	11730461	11936562	NA
jg10670	RGI	2	11751111	11758543	11936562	Putative threonine aspartase OS=Arabidopsis thaliana GN=At4g00590 PE=2 SV=3

jg10671	RGI	2	11782380	11783032	11936562	NA
jg10672	RGI	2	11786368	11787742	11936562	Early NAdulin-like protein 2 OS=Arabidopsis thaliana GN=At4g27520 PE=1 SV=1
jg10673	RGI	2	11806069	11806513	11936562	NA
jg10674	RGI	2	11808154	11808504	11936562	NA
jg10675	RGI	2	11812100	11814447	11936562	VQ motif-containing protein 4 OS=Arabidopsis thaliana GN=VQ4 PE=1 SV=1
jg10676	RGI	2	11843690	11849470	11936562	Dynamin-related protein 5A OS=Arabidopsis thaliana GN=DRP5A PE=2 SV=1
jg10677	RGI	2	11881676	11899440	11936562	Protein SPA1-RELATED 3 OS=Arabidopsis thaliana GN=SPA3 PE=1 SV=1
jg10678	RGI	2	11900996	11901450	11936562	NA
jg10679	RGI	2	11902700	11903727	11936562	NA
jg10680	RGI	2	11911842	11915747	11936562	NA
jg10681	RGI	2	11920453	11924880	11936562	Beta-glucuroNAsyltransferase GlcAT14A OS=Arabidopsis thaliana GN=GLCAT14A PE=2 SV=1
jg10682	RGI	2	11935244	11935462	11936562	NA
jg10683	RGI	2	11959302	11960266	11936562	NA
jg10684	RGI	2	11978496	11983686	11936562	Proton pump-interactor 1 OS=Arabidopsis thaliana GN=PPI1 PE=1 SV=2
jg10685	RGI	2	11999255	12003793	11936562	Chromatin remodeling protein EBS OS=Arabidopsis thaliana GN=EBS PE=1 SV=1
jg10686	RGI	2	12005709	12006053	11936562	NA
jg10687	RGI	2	12028634	12044115	11936562	Protein SAWADEE HOMEODOMAIN HOMOLOG 2 OS=Arabidopsis thaliana GN=SHH2 PE=2 SV=1
jg10688	RGI	2	12045048	12047290	11936562	NA
jg10689	RGI	2	12057214	12060969	11936562	NA
jg10690	RGI	2	12061012	12061284	11936562	NA
jg10691	RGI	2	12065979	12067101	11936562	RING-H2 finger protein ATL60 OS=Arabidopsis thaliana GN=ATL60 PE=2 SV=1
jg10692	RGI	2	12094247	12106452	11936562	NA
jg10693	RGI	2	12136124	12140499	11936562	3-deoxy-manNA-octulosonate cytidylyltransferase, mitochondrial OS=Arabidopsis thaliana GN=KDSB PE=1 SV=1
jg10694	RGI	2	12165613	12168242	11936562	NA

jg10695	RGI	2	12187152	12189525	11936562	E3 ubiquitin-protein ligase RMA3 OS=Arabidopsis thaliana GN=RMA3 PE=1 SV=1
jg10696	RGI	2	12203573	12206427	11936562	Thioredoxin M4, chloroplastic OS=Arabidopsis thaliana GN=At3g15360 PE=2 SV=2
jg9890	RGI	3	23745314	23745925	24016456	NA
jg9891	RGI	3	23777208	23779817	24016456	NA
jg9892	RGI	3	23784770	23785298	24016456	NA
jg9893	RGI	3	23789176	23791752	24016456	NA
jg9894	RGI	3	23791790	23792894	24016456	NA
jg9895	RGI	3	23816295	23816501	24016456	NA
jg9896	RGI	3	23816590	23818419	24016456	NA
jg9897	RGI	3	23826475	23827312	24016456	NA
jg9898	RGI	3	23831193	23831474	24016456	NA
jg9899	RGI	3	23840827	23845390	24016456	NA
jg9900	RGI	3	23880179	23883989	24016456	Gibberellin 2-beta-dioxygenase 8 OS=Arabidopsis thaliana GN=GA2OX8 PE=1 SV=2
jg9901	RGI	3	23946978	23948341	24016456	Protein NDR1 OS=Arabidopsis thaliana GN=NDR1 PE=1 SV=1
jg9902	RGI	3	23948674	23949482	24016456	GDSL esterase/lipase At3g26430 OS=Arabidopsis thaliana GN=At3g26430 PE=2 SV=1
jg9903	RGI	3	23985894	23993868	24016456	Probable lysine-specific demethylase JMJ14 OS=Arabidopsis thaliana GN=JMJ14 PE=1 SV=1
jg9904	RGI	3	24007152	24011878	24016456	NA
jg9905	RGI	3	24075154	24075638	24016456	NA
jg9906	RGI	3	24113707	24114168	24016456	NA
jg9907	RGI	3	24127747	24128157	24016456	NA
jg9908	RGI	3	24133793	24134102	24016456	NA
jg9909	RGI	3	24139643	24142409	24016456	NA
jg9910	RGI	3	24151934	24152568	24016456	NA
jg9911	RGI	3	24168218	24169151	24016456	NA
jg9912	RGI	3	24171463	24179855	24016456	Pentatricopeptide repeat-containing protein At4g04790, mitochondrial OS=Arabidopsis thaliana GN=At4g04790 PE=2 SV=2
jg9913	RGI	3	24180439	24180959	24016456	NA
jg9914	RGI	3	24181413	24182499	24016456	NA

jg9915	RGI	3	24205738	24209618	24016456	Polycomb group protein FERTILIZATION-INDEPENDENT ENDOSPERM OS=Arabidopsis thaliana GN=FIE PE=1 SV=2
jg9916	RGI	3	24210563	24222136	24016456	NA
jg9917	RGI	3	24248753	24249339	24016456	NA
jg9918	RGI	3	24249848	24250132	24016456	NA
jg9919	RGI	3	24269405	24275961	24016456	Autophagy-related protein 18f OS=Arabidopsis thaliana GN=ATG18F PE=2 SV=1
jg9920	RGI	3	24280229	24284506	24016456	NA
jg9921	RGI	3	24285771	24286019	24016456	NA
jg9922	RGI	3	24286075	24286509	24016456	NA
jg9923	RGI	3	24294716	24299783	24016456	Probable galacturoNAsyltransferase 12 OS=Arabidopsis thaliana GN=GAUT12 PE=2 SV=1
jg9924	RGI	3	24296189	24299783	24016456	Probable galacturoNAsyltransferase 12 OS=Arabidopsis thaliana GN=GAUT12 PE=2 SV=1
jg9925	RGI	3	24299801	24302354	24016456	Transcription factor ILR3 OS=Arabidopsis thaliana GN=ILR3 PE=1 SV=1
jg9926	RGI	3	24314798	24315188	24016456	NA
jg5699	RGI	4	33759434	33760223	34059117	NA
jg5700	RGI	4	33834284	33835146	34059117	NA
jg5701	RGI	4	33865721	33866954	34059117	NA
jg5702	RGI	4	33873434	33878086	34059117	Transcription factor MYB113 OS=Arabidopsis thaliana GN=MYB113 PE=1 SV=1
jg5703	RGI	4	33877875	33878175	34059117	NA
jg5704	RGI	4	33880254	33881242	34059117	Copper transporter 6 OS=Arabidopsis thaliana GN=COPT6 PE=2 SV=1
jg5705	RGI	4	33896330	33900051	34059117	Heme-binding-like protein At3g10130, chloroplastic OS=Arabidopsis thaliana GN=At3g10130 PE=1 SV=1
jg5706	RGI	4	33905779	33921599	34059117	NA
jg5707	RGI	4	33918026	33919154	34059117	Transcription factor bHLH155 OS=Arabidopsis thaliana GN=BHLH155 PE=1 SV=1
jg5708	RGI	4	33947361	33947816	34059117	NA
jg5709	RGI	4	33963649	33964761	34059117	Trypsin inhibitor A OS=Glycine max GN=KTI3 PE=1 SV=2
jg5710	RGI	4	34007199	34007477	34059117	NA
jg5711	RGI	4	34007622	34007927	34059117	NA

jg5712	RGI	4	34014973	34015359	34059117	NA
jg5713	RGI	4	34040459	34043873	34059117	NA
jg5714	RGI	4	34102429	34104113	34059117	NA
jg5715	RGI	4	34107412	34110570	34059117	MDIS1-interacting receptor like kinase 2 OS=Arabidopsis thaliana GN=MIK2 PE=1 SV=3
jg5716	RGI	4	34111675	34112086	34059117	NA
jg5717	RGI	4	34123034	34123246	34059117	NA
jg5718	RGI	4	34127540	34127815	34059117	NA
jg5719	RGI	4	34139360	34139928	34059117	NA
jg5720	RGI	4	34152953	34153876	34059117	Putative lipid-transfer protein DIR1 OS=Arabidopsis thaliana GN=DIR1 PE=1 SV=1
jg5721	RGI	4	34160099	34161222	34059117	Copper transporter 6 OS=Arabidopsis thaliana GN=COPT6 PE=2 SV=1
jg5722	RGI	4	34180070	34182345	34059117	NA
jg5723	RGI	4	34182413	34188520	34059117	NA
jg5724	RGI	4	34206171	34207217	34059117	17.8 kDa class I heat shock protein OS=Arabidopsis thaliana GN=HSP17.8 PE=1 SV=1
jg5725	RGI	4	34224185	34227102	34059117	NA
jg5726	RGI	4	34252021	34256631	34059117	NA
jg5727	RGI	4	34274100	34274330	34059117	NA
jg5728	RGI	4	34291615	34292420	34059117	NA
jg5729	RGI	4	34297913	34298231	34059117	NA
jg5730	RGI	4	34316790	34317388	34059117	NA
jg5731	RGI	4	34320030	34321244	34059117	NA
jg5732	RGI	4	34331678	34332592	34059117	NA
jg5733	RGI	4	34342659	34343907	34059117	NA
jg5734	RGI	4	34349393	34351198	34059117	NA
jg5987	RGI	4	39510803	39523641	39812897	NA
jg5988	RGI	4	39534515	39535463	39812897	NA
jg5989	RGI	4	39548805	39549664	39812897	NA
jg5990	RGI	4	39549865	39550185	39812897	NA
jg5991	RGI	4	39551163	39551813	39812897	NA
jg5992	RGI	4	39553064	39554242	39812897	NA
jg5993	RGI	4	39554661	39559447	39812897	NA

jg5994	RGI	4	39560523	39561051	39812897	NA
jg5995	RGI	4	39561231	39561842	39812897	NA
jg5996	RGI	4	39562353	39563942	39812897	NA
jg5997	RGI	4	39564057	39568346	39812897	NA
jg5998	RGI	4	39602806	39603231	39812897	NA
jg5999	RGI	4	39612039	39615538	39812897	NA
jg6000	RGI	4	39616798	39617666	39812897	NA
jg6001	RGI	4	39615767	39620127	39812897	Protein TPR3 OS=Oryza sativa subsp. japonica GN=TPR3 PE=1 SV=1
jg6002	RGI	4	39616798	39620127	39812897	Topless-related protein 1 OS=Arabidopsis thaliana GN=TPR1 PE=1 SV=3
jg6003	RGI	4	39620519	39620800	39812897	NA
jg6004	RGI	4	39641257	39644445	39812897	NA
jg6005	RGI	4	39645736	39646624	39812897	NA
jg6006	RGI	4	39651683	39652114	39812897	NA
jg6007	RGI	4	39666137	39666726	39812897	NA
jg6008	RGI	4	39691394	39692140	39812897	NA
jg6009	RGI	4	39786226	39787136	39812897	NA
jg6010	RGI	4	39828060	39828497	39812897	NA
jg6011	RGI	4	39892075	39892784	39812897	NA
jg6012	RGI	4	39966830	39971381	39812897	Pentatricopeptide repeat-containing protein At3g09060 OS=Arabidopsis thaliana GN=At3g09060 PE=2 SV=1
jg6013	RGI	4	39966830	39971381	39812897	Pentatricopeptide repeat-containing protein At3g09060 OS=Arabidopsis thaliana GN=At3g09060 PE=2 SV=1
jg6014	RGI	4	39980082	40000419	39812897	NA
jg6015	RGI	4	39999004	40000419	39812897	NA
jg6016	RGI	4	40003722	40004879	39812897	NA
jg6017	RGI	4	40005207	40008450	39812897	Protein NRT1/ PTR FAMILY 8.1 OS=Arabidopsis thaliana GN=NPF8.1 PE=1 SV=1
jg6018	RGI	4	40020307	40022696	39812897	NA
jg6019	RGI	4	40024774	40024974	39812897	NA
jg6020	RGI	4	40034564	40035151	39812897	NA
jg6021	RGI	4	40039665	40052665	39812897	NA
jg6022	RGI	4	40061826	40062026	39812897	NA
jg6023	RGI	4	40070325	40071415	39812897	NA

jg6024	RGI	4	40099842	40102989	39812897	Laccase-4 OS=Arabidopsis thaliana GN=IRX12 PE=2 SV=2
jg6025	RGI	4	40105545	40108927	39812897	Transcription factor DIVARICATA OS=Antirrhinum majus GN=DIVARICATA PE=2 SV=1
jg6011	RGI	4	39892075	39892784	40188515	NA
jg6012	RGI	4	39966830	39971381	40188515	Pentatricopeptide repeat-containing protein At3g09060 OS=Arabidopsis thaliana GN=At3g09060 PE=2 SV=1
jg6013	RGI	4	39966830	39971381	40188515	Pentatricopeptide repeat-containing protein At3g09060 OS=Arabidopsis thaliana GN=At3g09060 PE=2 SV=1
jg6014	RGI	4	39980082	40000419	40188515	NA
jg6015	RGI	4	39999004	40000419	40188515	NA
jg6016	RGI	4	40003722	40004879	40188515	NA
jg6017	RGI	4	40005207	40008450	40188515	Protein NRT1/ PTR FAMILY 8.1 OS=Arabidopsis thaliana GN=NPF8.1 PE=1 SV=1
jg6018	RGI	4	40020307	40022696	40188515	NA
jg6019	RGI	4	40024774	40024974	40188515	NA
jg6020	RGI	4	40034564	40035151	40188515	NA
jg6021	RGI	4	40039665	40052665	40188515	NA
jg6022	RGI	4	40061826	40062026	40188515	NA
jg6023	RGI	4	40070325	40071415	40188515	NA
jg6024	RGI	4	40099842	40102989	40188515	Laccase-4 OS=Arabidopsis thaliana GN=IRX12 PE=2 SV=2
jg6025	RGI	4	40105545	40108927	40188515	Transcription factor DIVARICATA OS=Antirrhinum majus GN=DIVARICATA PE=2 SV=1
jg6026	RGI	4	40115462	40116240	40188515	NA
jg6027	RGI	4	40141497	40147459	40188515	SulfoquiNAvosyl transferase SQD2 OS=Arabidopsis thaliana GN=SQD2 PE=1 SV=1
jg6028	RGI	4	40154596	40156182	40188515	NA
jg6029	RGI	4	40180207	40182140	40188515	NA
jg6030	RGI	4	40184370	40199700	40188515	NA
jg6031	RGI	4	40205864	40206707	40188515	NA
jg6032	RGI	4	40289106	40290388	40188515	NA
jg6033	RGI	4	40325815	40329549	40188515	Glycerol-3-phosphate 2-O-acyltransferase 6 OS=Arabidopsis thaliana GN=GPAT6 PE=1 SV=1
jg6034	RGI	4	40332130	40333237	40188515	NA
jg6035	RGI	4	40338932	40339345	40188515	NA

jg6036	RGI	4	40339640	40339921	40188515	NA
jg6037	RGI	4	40413557	40417306	40188515	Probable leucine-rich repeat receptor-like protein kinase At1g35710 OS=Arabidopsis thaliana GN=At1g35710 PE=2 SV=1
jg6038	RGI	4	40422818	40423862	40188515	NA
jg6039	RGI	4	40425443	40431745	40188515	NA
jg6040	RGI	4	40491547	40493490	40188515	NA
jg6041	RGI	4	40504020	40506676	40188515	NA
jg6042	RGI	4	40517490	40519079	40188515	Probable aquaporin PIP1-4 OS=Arabidopsis thaliana GN=PIP1.4 PE=1 SV=1
jg6043	RGI	4	40531501	40533447	40188515	NA
jg6044	RGI	4	40532093	40538658	40188515	NA
jg6063	RGI	4	40765263	40766961	41058948	NA
jg6064	RGI	4	40768638	40770101	41058948	Uncharacterized protein At4g19900 OS=Arabidopsis thaliana GN=At4g19900 PE=2 SV=1
jg6065	RGI	4	40772857	40773647	41058948	NA
jg6066	RGI	4	40784515	40788180	41058948	Probable LRR receptor-like serine/threonine-protein kinase At3g47570 OS=Arabidopsis thaliana GN=At3g47570 PE=2 SV=1
jg6067	RGI	4	40796250	40797571	41058948	NA
jg6068	RGI	4	40797608	40798849	41058948	NA
jg6069	RGI	4	40808131	40809700	41058948	NA
jg6071	RGI	4	40823689	40830788	41058948	RNA polymerase II C-terminal domain phosphatase-like 2 OS=Arabidopsis thaliana GN=CPL2 PE=1 SV=3
jg6072	RGI	4	40838514	40844344	41058948	Cold-responsive protein kinase 1 OS=Arabidopsis thaliana GN=CRPK1 PE=1 SV=1
jg6073	RGI	4	40843379	40846476	41058948	NA
jg6074	RGI	4	40861816	40866027	41058948	NA
jg6075	RGI	4	40876395	40880592	41058948	Protein yippee-like OS=Solanum tuberosum PE=2 SV=1
jg6076	RGI	4	40877756	40880592	41058948	Protein yippee-like OS=Solanum tuberosum PE=2 SV=1
jg6077	RGI	4	40901531	40905097	41058948	Probable LRR receptor-like serine/threonine-protein kinase At3g47570 OS=Arabidopsis thaliana GN=At3g47570 PE=2 SV=1
jg6079	RGI	4	40927332	40932374	41058948	E3 ubiquitin-protein ligase AIRP2 OS=Arabidopsis thaliana GN=AIRP2 PE=1 SV=1
jg6080	RGI	4	40959284	40965885	41058948	Protein root UVB sensitive 5 OS=Arabidopsis thaliana GN=RUS5 PE=2 SV=1

jg6081	RGI	4	40995214	41005051	41058948	Alcohol dehydrogenase-like 3 OS=Arabidopsis thaliana GN=At1g32780 PE=2 SV=1
jg6082	RGI	4	41008549	41008794	41058948	NA
jg6083	RGI	4	41011176	41015150	41058948	Probable diamiNApimelate decarboxylase, chloroplastic OS=Oryza sativa subsp. japonica GN=LYSA PE=2 SV=1
jg6084	RGI	4	41021178	41022520	41058948	NA
jg6085	RGI	4	41032062	41036402	41058948	Thylakoid ADP,ATP carrier protein, chloroplastic OS=Arabidopsis thaliana GN=TAAC PE=1 SV=1
jg6086	RGI	4	41041546	41049812	41058948	Vacuolar cation/proton exchanger 3 OS=Arabidopsis thaliana GN=CAX3 PE=1 SV=1
jg6087	RGI	4	41058322	41058648	41058948	NA
jg6088	RGI	4	41095487	41100483	41058948	LIMR family protein At5g01460 OS=Arabidopsis thaliana GN=At5g01460 PE=2 SV=1
jg6089	RGI	4	41126953	41128921	41058948	GDSL esterase/lipase CPRD49 OS=Arabidopsis thaliana GN=CPRD49 PE=2 SV=1
jg6090	RGI	4	41135313	41139653	41058948	NA
jg6091	RGI	4	41153341	41155249	41058948	NA
jg6092	RGI	4	41164151	41166442	41058948	NA
jg6093	RGI	4	41177103	41179061	41058948	Zeatin O-glucosyltransferase OS=Phaseolus lunatus GN=ZOG1 PE=2 SV=1
jg6094	RGI	4	41177103	41179298	41058948	Zeatin O-glucosyltransferase OS=Phaseolus lunatus GN=ZOG1 PE=2 SV=1
jg6096	RGI	4	41233753	41234557	41058948	NA
jg6097	RGI	4	41245430	41252912	41058948	NA
jg6098	RGI	4	41253875	41256108	41058948	NA
jg6099	RGI	4	41264138	41266101	41058948	NA
jg6100	RGI	4	41270086	41272092	41058948	GDSL esterase/lipase CPRD49 OS=Arabidopsis thaliana GN=CPRD49 PE=2 SV=1
jg6101	RGI	4	41283988	41285975	41058948	Probable protein phosphatase 2C 73 OS=Arabidopsis thaliana GN=PPC6-7 PE=2 SV=1
jg6102	RGI	4	41286141	41289689	41058948	GDSL esterase/lipase CPRD49 OS=Arabidopsis thaliana GN=CPRD49 PE=2 SV=1
jg6103	RGI	4	41319150	41321262	41058948	NA
jg6104	RGI	4	41332599	41332883	41058948	NA

jg6105	RGI	4	41353001	41353216	41058948	NA
jg6106	RGI	4	41353289	41353504	41058948	NA
jg6107	RGI	4	41357006	41357461	41058948	NA
jg23079	RGI	7	36929063	36929597	37173579	NA
jg23080	RGI	7	36937322	36940181	37173579	Cyclin-D4-1 OS=Oryza sativa subsp. japonica GN=CYCD4-1 PE=2 SV=2
jg23081	RGI	7	36943888	36944215	37173579	NA
jg23082	RGI	7	36971381	36972712	37173579	NA
jg23083	RGI	7	37010566	37011009	37173579	NA
jg23084	RGI	7	37064499	37074698	37173579	ATP-dependent 6-phosphofructokinase 5, chloroplastic OS=Arabidopsis thaliana GN=PFK5 PE=1 SV=1
jg23085	RGI	7	37099278	37099493	37173579	NA
jg23086	RGI	7	37125853	37127827	37173579	Zinc-finger homeodomain protein 1 OS=Oryza sativa subsp. japonica GN=ZHD1 PE=2 SV=1
jg23087	RGI	7	37131739	37132200	37173579	NA
jg23088	RGI	7	37274253	37292312	37173579	GLABRA2 expression modulator OS=Arabidopsis thaliana GN=GEM PE=1 SV=1
jg23089	RGI	7	37308881	37312579	37173579	NA
jg23090	RGI	7	37317312	37317992	37173579	NA
jg23091	RGI	7	37392315	37397121	37173579	NA
jg23092	RGI	7	37411613	37414941	37173579	NA
jg23093	RGI	7	37433621	37434162	37173579	NA
jg23094	RGI	7	37434212	37434838	37173579	NA
jg23095	RGI	7	37455903	37459491	37173579	BTB/POZ domain-containing protein At1g67900 OS=Arabidopsis thaliana GN=At1g67900 PE=1 SV=1
jg23096	RGI	7	37461266	37462433	37173579	NA
jg23097	RGI	7	37464690	37471175	37173579	NA
jg23989	RGI	7	55894774	55895812	56140780	NA
jg23990	RGI	7	55906667	55910112	56140780	NA
jg23991	RGI	7	55925869	55927591	56140780	NA
jg23992	RGI	7	55957885	55958544	56140780	NA
jg23993	RGI	7	55958748	55959249	56140780	NA
jg23994	RGI	7	55974109	55976654	56140780	NA
jg23995	RGI	7	56003258	56003899	56140780	NA

jg23996	RGI	7	56037074	56037289	56140780	NA
jg23997	RGI	7	56056998	56057374	56140780	NA
jg23998	RGI	7	56068451	56070029	56140780	NA
jg23999	RGI	7	56122042	56125274	56140780	NA
jg24000	RGI	7	56166068	56171886	56140780	NA
jg24001	RGI	7	56177142	56186869	56140780	Berberine bridge enzyme-like 23 OS=Arabidopsis thaliana GN=At5g44360 PE=2 SV=1
jg24002	RGI	7	56230256	56247256	56140780	NA
jg24003	RGI	7	56274889	56276324	56140780	NA
jg24004	RGI	7	56292353	56292595	56140780	NA
jg24005	RGI	7	56307151	56310488	56140780	NA
jg24006	RGI	7	56379124	56380002	56140780	NA
jg24007	RGI	7	56388714	56389583	56140780	NA
jg24008	RGI	7	56390173	56392258	56140780	NA
jg24009	RGI	7	56392661	56393005	56140780	NA
jg24010	RGI	7	56394024	56394353	56140780	NA
jg24011	RGI	7	56394838	56395098	56140780	NA
jg24012	RGI	7	56395546	56395809	56140780	NA
jg24013	RGI	7	56409228	56411266	56140780	NA
jg24014	RGI	7	56411513	56412022	56140780	NA
jg24015	RGI	7	56427593	56428018	56140780	NA
jg24016	RGI	7	56428851	56431173	56140780	E3 ubiquitin-protein ligase SINA-like 10 OS=Arabidopsis thaliana GN=At5g37930 PE=2 SV=1
jg37444	RGI	8	22450530	22452580	22721042	Transcription factor RAX2 OS=Arabidopsis thaliana GN=RAX2 PE=1 SV=1
jg37445	RGI	8	22466778	22468373	22721042	NA
jg37446	RGI	8	22481840	22482175	22721042	NA
jg37447	RGI	8	22485480	22486123	22721042	NA
jg37448	RGI	8	22491342	22498568	22721042	Protein PTST homolog 3, chloroplastic OS=Arabidopsis thaliana GN=PTST PE=1 SV=1
jg37449	RGI	8	22501937	22503945	22721042	NA
jg37450	RGI	8	22506782	22507105	22721042	NA
jg37451	RGI	8	22518716	22522038	22721042	NA

jg37452	RGI	8	22518716	22519281	22721042	CHD3-type chromatin-remodeling factor PICKLE OS=Arabidopsis thaliana GN=PKL PE=1 SV=1
jg37453	RGI	8	22527647	22528536	22721042	NA
jg37454	RGI	8	22540639	22541503	22721042	NA
jg37455	RGI	8	22545402	22547763	22721042	S-adeNAsylmethionine synthase 1 OS=Vitis vinifera GN=METK1 PE=3 SV=1
jg37456	RGI	8	22553600	22554135	22721042	NA
jg37457	RGI	8	22558772	22559080	22721042	NA
jg37458	RGI	8	22604406	22610034	22721042	DEAD-box ATP-dependent RNA helicase 13 OS=Oryza sativa subsp. japonica GN=Os04g0510400 PE=2 SV=2
jg37459	RGI	8	22614577	22616172	22721042	NA
jg37460	RGI	8	22623460	22625838	22721042	NA
jg37461	RGI	8	22628177	22639969	22721042	Transcription factor EMB1444 OS=Arabidopsis thaliana GN=EMB1444 PE=2 SV=1
jg37462	RGI	8	22647478	22648311	22721042	NA
jg37463	RGI	8	22662093	22664415	22721042	WAT1-related protein At4g08290 OS=Arabidopsis thaliana GN=At4g08290 PE=2 SV=1
jg37464	RGI	8	22664499	22667822	22721042	CDT1-like protein a, chloroplastic OS=Arabidopsis thaliana GN=CDT1A PE=1 SV=1
jg37465	RGI	8	22673828	22674537	22721042	NA
jg37466	RGI	8	22679727	22679969	22721042	NA
jg37467	RGI	8	22680097	22680768	22721042	NA
jg37468	RGI	8	22700646	22708003	22721042	Autophagy-related protein 9 OS=Arabidopsis thaliana GN=ATG9 PE=2 SV=1
jg37469	RGI	8	22736538	22741080	22721042	Protein KINESIN LIGHT CHAIN-RELATED 2 OS=Arabidopsis thaliana GN=KLCR2 PE=1 SV=1
jg37470	RGI	8	22742015	22743922	22721042	RING-H2 finger protein ATL1 OS=Arabidopsis thaliana GN=ATL1 PE=2 SV=1
jg37471	RGI	8	22745172	22747045	22721042	Protein DOWNY MILDEW RESISTANCE 6 OS=Arabidopsis thaliana GN=DMR6 PE=1 SV=1
jg37472	RGI	8	22748189	22748639	22721042	NA
jg37473	RGI	8	22761271	22761588	22721042	NA
jg37474	RGI	8	22777947	22778630	22721042	NA
jg37475	RGI	8	22780368	22786226	22721042	NA

jg37476	RGI	8	22787164	22788495	22721042	NA
jg37477	RGI	8	22792512	22792934	22721042	NA
jg37478	RGI	8	22830846	22831435	22721042	NA
jg37479	RGI	8	22832761	22833405	22721042	NA
jg37480	RGI	8	22862209	22867132	22721042	NA
jg37481	RGI	8	22868351	22891133	22721042	Structural maintenance of chromosomes protein 1 OS=Arabidopsis thaliana GN=SMC1 PE=2 SV=2
jg37482	RGI	8	22904664	22905200	22721042	Probable methyltransferase PMT16 OS=Arabidopsis thaliana GN=At2g45750 PE=3 SV=1
jg37483	RGI	8	22914885	22915699	22721042	Ethylene-responsive transcription factor 1B OS=Arabidopsis thaliana GN=ERF1B PE=1 SV=2
jg37484	RGI	8	22924908	22925703	22721042	Ethylene-responsive transcription factor 1B OS=Arabidopsis thaliana GN=ERF1B PE=1 SV=2
jg37485	RGI	8	22946909	22947121	22721042	NA
jg37486	RGI	8	22955659	22955922	22721042	NA
jg37487	RGI	8	22962288	22962569	22721042	NA
jg37488	RGI	8	22963649	22966152	22721042	NA
jg37489	RGI	8	22981831	22982602	22721042	Ethylene-responsive transcription factor 1B OS=Arabidopsis thaliana GN=ERF1B PE=1 SV=2
jg37490	RGI	8	23015889	23016273	22721042	NA
jg37491	RGI	8	23017787	23018368	22721042	Ethylene-responsive transcription factor ERF098 OS=Arabidopsis thaliana GN=ERF098 PE=1 SV=1
jg38796	RGI	8	38211083	38215630	38508969	Protein SIEVE ELEMENT OCCLUSION B OS=Arabidopsis thaliana GN=SEOB PE=1 SV=1
jg38797	RGI	8	38220844	38221871	38508969	PhosphoeNAlpyruvate carboxylase kinase 2 OS=Arabidopsis thaliana GN=PPCK2 PE=1 SV=2
jg38798	RGI	8	38223825	38225752	38508969	NA
jg38799	RGI	8	38229242	38229574	38508969	NA
jg38800	RGI	8	38232956	38233198	38508969	NA
jg38801	RGI	8	38234051	38235868	38508969	Probable iNArganic phosphate transporter 1-7 OS=Arabidopsis thaliana GN=PHT1-7 PE=2 SV=2
jg38802	RGI	8	38240211	38243869	38508969	INArganic phosphate transporter 1-4 OS=Arabidopsis thaliana GN=PHT1-4 PE=1 SV=1

jg38803	RGI	8	38256927	38261537	38508969	Serine/threonine-protein phosphatase PP1 OS=Oryza sativa subsp. japonica GN=Os03g0268000 PE=2 SV=2
jg38804	RGI	8	38263487	38266384	38508969	NA
jg38805	RGI	8	38270134	38271892	38508969	NA
jg38806	RGI	8	38282880	38283092	38508969	NA
jg38807	RGI	8	38285451	38287070	38508969	Ethylene-responsive transcription factor 1B OS=Arabidopsis thaliana GN=ERF1B PE=1 SV=2
jg38808	RGI	8	38318963	38319647	38508969	Ethylene-responsive transcription factor ERF098 OS=Arabidopsis thaliana GN=ERF098 PE=1 SV=1
jg38809	RGI	8	38335754	38336793	38508969	Ethylene-responsive transcription factor ERF096 OS=Arabidopsis thaliana GN=ERF096 PE=1 SV=1
jg38810	RGI	8	38345483	38347534	38508969	RING-H2 finger protein ATL16 OS=Arabidopsis thaliana GN=ATL16 PE=2 SV=1
jg38811	RGI	8	38351968	38355557	38508969	Electron transfer flavoprotein subunit beta, mitochondrial OS=Arabidopsis thaliana GN=ETFB PE=1 SV=1
jg38812	RGI	8	38360348	38362589	38508969	WAT1-related protein At5g07050 OS=Arabidopsis thaliana GN=At5g07050 PE=2 SV=1
jg38813	RGI	8	38370661	38373861	38508969	CASP-like protein 5B3 OS=Arabidopsis thaliana GN=At3g23200 PE=2 SV=1
jg38814	RGI	8	38385078	38397040	38508969	Probable protein phosphatase 2C 22 OS=Arabidopsis thaliana GN=At2g25620 PE=1 SV=1
jg38815	RGI	8	38385078	38397040	38508969	Probable protein phosphatase 2C 22 OS=Arabidopsis thaliana GN=At2g25620 PE=1 SV=1
jg38816	RGI	8	38385078	38397040	38508969	Homogentisate phytyltransferase 1, chloroplastic OS=Arabidopsis thaliana GN=HPT1 PE=1 SV=1
jg38817	RGI	8	38401723	38404704	38508969	CASP-like protein 5B3 OS=Arabidopsis thaliana GN=At3g23200 PE=2 SV=1
jg38818	RGI	8	38409519	38410064	38508969	NA
jg38819	RGI	8	38412682	38415116	38508969	NA
jg38820	RGI	8	38415468	38418459	38508969	NA
jg38821	RGI	8	38424292	38436805	38508969	Actin-related protein 9 OS=Arabidopsis thaliana GN=ARP9 PE=2 SV=1
jg38822	RGI	8	38443067	38444456	38508969	Uncharacterized protein At4g14450, chloroplastic OS=Arabidopsis thaliana GN=At4g14450 PE=2 SV=1

jg38823	RGI	8	38451005	38453594	38508969	NA
jg38824	RGI	8	38460605	38465702	38508969	NA
jg38825	RGI	8	38475774	38478195	38508969	NA
jg38826	RGI	8	38484012	38488394	38508969	Ethylene receptor 2 OS=Arabidopsis thaliana GN=ETR2 PE=1 SV=2
jg38827	RGI	8	38484012	38488394	38508969	Ethylene receptor 2 OS=Arabidopsis thaliana GN=ETR2 PE=1 SV=2
jg38828	RGI	8	38519079	38536147	38508969	TNF receptor-associated factor homolog 1b OS=Arabidopsis thaliana GN=TRAF1B PE=1 SV=1
jg38829	RGI	8	38542672	38543200	38508969	NA
jg38830	RGI	8	38547898	38548690	38508969	NA
jg38831	RGI	8	38552747	38553439	38508969	NA
jg38832	RGI	8	38556025	38558807	38508969	Protein PIN-LIKES 3 OS=Arabidopsis thaliana GN=PILS3 PE=2 SV=1
jg38833	RGI	8	38559833	38564522	38508969	Protein unc-13 homolog OS=Arabidopsis thaliana GN=PATROL1 PE=2 SV=1
jg38834	RGI	8	38565333	38569148	38508969	Cellulose synthase-like protein G2 OS=Arabidopsis thaliana GN=CSLG2 PE=2 SV=1
jg38836	RGI	8	38587802	38591769	38508969	Heterogeneous nuclear ribonucleoprotein 1 OS=Arabidopsis thaliana GN=RNP1 PE=1 SV=1
jg38837	RGI	8	38596742	38599804	38508969	NA
jg38838	RGI	8	38596742	38601103	38508969	NA
jg38839	RGI	8	38606024	38609038	38508969	NA
jg38840	RGI	8	38619215	38620814	38508969	NA
jg38841	RGI	8	38627675	38631134	38508969	Uncharacterized protein At5g64816 OS=Arabidopsis thaliana GN=At5g64816 PE=2 SV=1
jg38842	RGI	8	38632540	38636564	38508969	NA
jg38843	RGI	8	38632540	38637004	38508969	NA
jg38844	RGI	8	38642650	38645115	38508969	DELLA protein GAI OS=Arabidopsis thaliana GN=GAI PE=1 SV=1
jg38845	RGI	8	38645265	38649263	38508969	Zinc finger protein CONSTANS-LIKE 4 OS=Arabidopsis thaliana GN=COL4 PE=2 SV=2
jg38847	RGI	8	38667380	38669497	38508969	Cysteine-rich repeat secretory protein 3 OS=Arabidopsis thaliana GN=CRRSP3 PE=1 SV=1
jg38848	RGI	8	38671438	38675097	38508969	Pentatricopeptide repeat-containing protein At1g25360 OS=Arabidopsis thaliana GN=PCMP-H74 PE=2 SV=1
jg38849	RGI	8	38671866	38675097	38508969	Pentatricopeptide repeat-containing protein At1g25360 OS=Arabidopsis thaliana GN=PCMP-H74 PE=2 SV=1

jg38850	RGI	8	38675833	38679464	38508969	NA
jg38852	RGI	8	38688182	38692167	38508969	NA
jg38853	RGI	8	38692628	38695638	38508969	3-isopropylmalate dehydratase large subunit, chloroplastic OS=Arabidopsis thaliana GN=IIL1 PE=1 SV=1
jg38854	RGI	8	38692628	38698132	38508969	3-isopropylmalate dehydratase large subunit, chloroplastic OS=Arabidopsis thaliana GN=IIL1 PE=1 SV=1
jg38855	RGI	8	38692628	38698648	38508969	3-isopropylmalate dehydratase large subunit, chloroplastic OS=Arabidopsis thaliana GN=IIL1 PE=1 SV=1
jg38856	RGI	8	38700752	38704055	38508969	NA
jg38857	RGI	8	38703960	38706981	38508969	NA
jg38858	RGI	8	38709716	38710202	38508969	Ripening-related protein grip22 OS=Vitis vinifera GN=grip22 PE=2 SV=1
jg38859	RGI	8	38712719	38713072	38508969	NA
jg38860	RGI	8	38715907	38716377	38508969	NA
jg38861	RGI	8	38727202	38727709	38508969	NA
jg38862	RGI	8	38727512	38728235	38508969	NA
jg38863	RGI	8	38732999	38734087	38508969	Transcription factor MYB2 OS=Oryza sativa subsp. japonica GN=MYB2 PE=2 SV=1
jg38864	RGI	8	38738756	38741468	38508969	Pentatricopeptide repeat-containing protein At3g57430, chloroplastic OS=Arabidopsis thaliana GN=PCMP-H81 PE=2 SV=2
jg38865	RGI	8	38739028	38741468	38508969	Pentatricopeptide repeat-containing protein At3g57430, chloroplastic OS=Arabidopsis thaliana GN=PCMP-H81 PE=2 SV=2
jg38866	RGI	8	38747365	38751510	38508969	Haloacid dehalogenase-like hydrolase domain-containing protein At2g33255 OS=Arabidopsis thaliana GN=At2g33255 PE=1 SV=1
jg38867	RGI	8	38756025	38762048	38508969	Carboxyl-terminal-processing peptidase 2, chloroplastic OS=Arabidopsis thaliana GN=CTPA2 PE=1 SV=1
jg38868	RGI	8	38762780	38769971	38508969	Polyadenylate-binding protein 1 OS=Arabidopsis thaliana GN=PABN1 PE=1 SV=1
jg38869	RGI	8	38783186	38786052	38508969	Cyclin-dependent kinase inhibitor 3 OS=Arabidopsis thaliana GN=KRP3 PE=1 SV=1
jg38870	RGI	8	38793363	38798063	38508969	NA
jg38872	RGI	8	38809742	38831741	38508969	ATP-dependent DNA helicase SRS2-like protein At4g25120 OS=Arabidopsis thaliana GN=SRS2 PE=1 SV=1

jg32966	RGI	10	33092650	33094965	33340353	E3 ubiquitin-protein ligase RZF1 OS=Arabidopsis thaliana GN=RZF1 PE=1 SV=1
jg32967	RGI	10	33095142	33095527	33340353	NA
jg32968	RGI	10	33120098	33122594	33340353	NA
jg32969	RGI	10	33145691	33148538	33340353	NA
jg32970	RGI	10	33153738	33154112	33340353	NA
jg32971	RGI	10	33194573	33196760	33340353	NA
jg32972	RGI	10	33197649	33212008	33340353	Proline-rich receptor-like protein kinase PERK15 OS=Arabidopsis thaliana GN=PERK15 PE=2 SV=1
jg32973	RGI	10	33220622	33223211	33340353	NA
jg32974	RGI	10	33239060	33240560	33340353	Zinc finger protein ZAT2 OS=Arabidopsis thaliana GN=ZAT2 PE=1 SV=1
jg32975	RGI	10	33262755	33263068	33340353	NA
jg32976	RGI	10	33296274	33296916	33340353	NA
jg32977	RGI	10	33330470	33330877	33340353	NA
jg32978	RGI	10	33338372	33339160	33340353	NA
jg32979	RGI	10	33354249	33357312	33340353	NA
jg32980	RGI	10	33379807	33380127	33340353	NA
jg32981	RGI	10	33438056	33439622	33340353	NA
jg32982	RGI	10	33498519	33514201	33340353	NA
jg32983	RGI	10	33537255	33538090	33340353	NA
jg32984	RGI	10	33555228	33556362	33340353	Cellulose synthase A catalytic subunit 7 [UDP-forming] OS=Oryza sativa subsp. japonica GN=CESA7 PE=2 SV=1
jg32985	RGI	10	33556635	33609389	33340353	Nudix hydrolase 3 OS=Arabidopsis thaliana GN=NUDT3 PE=1 SV=1
jg32986	RGI	10	33619005	33623636	33340353	FlavoNAid 3',5'-hydroxylase 2 OS=Petunia hybrida GN=CYP75A3 PE=2 SV=1
