# Supplementary material for: Integration of GWAS and RNA-Seq Analysis to Identify SNPs and Candidate Genes Associated with Alkali Stress Tolerance at the Germination Stage in Mung Bean
Source: Genes (Basel). 2023 Jun 19;14(6):1294. doi: 10.3390/genes14061294 (PMC10298294; doi:10.3390/genes14061294)
Supplement: Supplementary file 1 [file genes-14-01294-s001.zip › Supplementary Materials/Table S5. Statistics of sequencing data quality by RNA-seq.pdf]

Table S5. Statistics of sequencing data quality by RNA-seq.

Samples	Raw reads	Clean reads	Q20(%)	Q30(%)	GC content(%)	Total mapped reads	unique mapped reads	multiple mapped reads
TM1	42319120	40882408	97.55	93.11	44.85	39100599(95.64%)	38164915(93.35%)	935684(2.29%)
TM2	46418080	45572948	97.52	93.08	44.71	43990375(96.53%)	42949968(94.24%)	1040407(2.28%)
TM3	45178936	44217212	97.67	93.42	44.50	42750591(96.68%)	41693422(94.29%)	1057169(2.39%)
TMT1	46634424	45953878	97.62	93.25	44.21	44376133(96.57%)	43352859(94.34%)	1023274(2.23%)
TMT2	42090322	40133988	97.65	93.43	45.11	38171509(95.11%)	37342806(93.05%)	828703(2.06%)
TMT3	44671390	42794062	97.73	93.56	44.77	40614789(94.91%)	39759979(92.91%)	854810(2.0%)
