# Supplementary material for: Integration of GWAS and RNA-Seq Analysis to Identify SNPs and Candidate Genes Associated with Alkali Stress Tolerance at the Germination Stage in Mung Bean
Source: Genes (Basel). 2023 Jun 19;14(6):1294. doi: 10.3390/genes14061294 (PMC10298294; doi:10.3390/genes14061294)
Supplement: Supplementary file 1 [file genes-14-01294-s001.zip › Supplementary Materials/Table S6. Gene Ontology (GO) analysis for 2565 DEGs.pdf]

Table S6. Gene Ontology (GO) analysis for 2565 DEGs.

Category	GOID	Description	Gene Ratio	Bg Ratio	*P* value	Padj	Gene ID
BP	GO:0006979	response to oxidative stress	21/655	80/6148	6.33E-05	0.012066932	novel.229/jg16891/jg31038/novel.230/jg8118/novel.454/novel.737/novel.261/novel.116/jg9023/jg20275/jg11952/jg38691/jg33679/jg31699/novel.85/jg2948/jg30160/jg3461/jg38233/jg4794
BP	GO:0071554	cell wall organization or biogenesis	19/655	69/6148	6.95E-05	0.012066932	jg34074/jg1307/jg14987/jg29753/jg14986/jg21946/jg38108/jg12654/jg1316/jg3376/jg38109/jg3375/jg21947/jg21321/jg31535/jg3374/jg26874/jg33498/jg21951
BP	GO:0006950	response to stress	45/655	242/6148	0.000112876	0.012066932	jg20016/jg20015/jg24134/jg24133/novel.229/jg16891/jg24137/jg31038/novel.230/jg8118/novel.454/novel.737/jg29637/novel.261/novel.116/jg10452/jg20017/jg17863/jg9023/jg20275/jg39227/jg12934/jg25997/jg24420/jg39848/jg3030/jg11952/jg38077/jg8783/jg38691/jg4986/jg33679/jg31699/novel.85/jg34122/jg2948/jg20013/jg38775/jg30160/jg8288/jg20011/jg3461/jg27441/jg38233/jg4794
BP	GO:0006026	aminoglycan catabolic process	6/655	11/6148	0.000412545	0.012066932	jg34074/jg29753/jg21946/jg12654/jg21947/jg21951
BP	GO:0006030	chitin metabolic process	6/655	11/6148	0.000412545	0.012066932	jg34074/jg29753/jg21946/jg12654/jg21947/jg21951
BP	GO:0006032	chitin catabolic process	6/655	11/6148	0.000412545	0.012066932	jg34074/jg29753/jg21946/jg12654/jg21947/jg21951
BP	GO:0006040	amino sugar metabolic process	6/655	11/6148	0.000412545	0.012066932	jg34074/jg29753/jg21946/jg12654/jg21947/jg21951
BP	GO:0016998	cell wall macromolecule catabolic process	6/655	11/6148	0.000412545	0.012066932	jg34074/jg29753/jg21946/jg12654/jg21947/jg21951
BP	GO:0044036	cell wall macromolecule metabolic process	6/655	11/6148	0.000412545	0.012066932	jg34074/jg29753/jg21946/jg12654/jg21947/jg21951

BP	GO:0046348	amino sugar catabolic process	6/655	11/6148	0.000412545	0.012066932	jg34074/jg29753/jg21946/jg12654/jg21947/jg21951
BP	GO:1901071	glucosamine-containing compound metabolic process	6/655	11/6148	0.000412545	0.012066932	jg34074/jg29753/jg21946/jg12654/jg21947/jg21951
BP	GO:1901072	glucosamine-containing compound catabolic process	6/655	11/6148	0.000412545	0.012066932	jg34074/jg29753/jg21946/jg12654/jg21947/jg21951
BP	GO:0009607	response to biotic stimulus	10/655	31/6148	0.00097141	0.026228074	jg20016/jg20015/jg24134/jg24133/jg24137/jg10452/jg20017/jg25997/jg20013/jg20011
BP	GO:0006952	defense response	13/655	50/6148	0.0017141	0.042974939	jg20016/jg20015/jg24134/jg24133/jg24137/jg10452/jg20017/jg25997/jg3030/jg34122/jg20013/jg20011/jg27441
BP	GO:0006022	aminoglycan metabolic process	6/655	14/6148	0.002024305	0.044408181	jg34074/jg29753/jg21946/jg12654/jg21947/jg21951
BP	GO:0042737	drug catabolic process	6/655	14/6148	0.002024305	0.044408181	jg34074/jg29753/jg21946/jg12654/jg21947/jg21951
MF	GO:0004601	peroxidase activity	23/960	86/9398	1.09E-05	0.001972045	jg5488/novel.229/jg16891/jg31038/novel.230/jg8118/novel.454/novel.737/novel.261/novel.116/jg29555/jg9023/jg20275/jg11952/jg38691/jg33679/jg31699/novel.85/jg2948/jg30160/jg3461/jg38233/jg4794
MF	GO:0016684	oxidoreductase activity, acting on peroxide as acceptor	23/960	88/9398	1.65E-05	0.001972045	jg5488/novel.229/jg16891/jg31038/novel.230/jg8118/novel.454/novel.737/novel.261/novel.116/jg29555/jg9023/jg20275/jg11952/jg38691/jg33679/jg31699/novel.85/jg2948/jg30160/jg3461/jg38233/jg4794
MF	GO:0016209	antioxidant activity	23/960	93/9398	4.31E-05	0.003430342	jg5488/novel.229/jg16891/jg31038/novel.230/jg8118/novel.454/novel.737/novel.261/novel.116/jg29555/jg9023/jg20275/jg11952/jg38691/jg33679/jg31699/novel.85/jg2948/jg30160/jg3461/jg38233/jg4794

MF	GO:0020037	heme binding	46/960	266/9398	0.000231002	0.012060609	jg33801/novel.229/jg16891/jg2226/jg31038/novel.230/jg8118/novel.454/novel.737/jg33957/novel.261/jg22104/novel.116/jg29555/jg2167/jg8457/jg12924/jg9023/jg20275/jg8041/jg22392/jg39164/jg11952/jg2130/jg38691/jg26106/jg23482/jg33679/jg31699/jg4193/novel.455/novel.85/jg2517/jg33261/jg8043/jg4192/jg2948/jg30160/jg23117/novel.490/jg26107/jg26105/jg3461/jg38233/jg4794/jg31098
MF	GO:0046906	tetrapyrrole binding	46/960	267/9398	0.000252314	0.012060609	jg33801/novel.229/jg16891/jg2226/jg31038/novel.230/jg8118/novel.454/novel.737/jg33957/novel.261/jg22104/novel.116/jg29555/jg2167/jg8457/jg12924/jg9023/jg20275/jg8041/jg22392/jg39164/jg11952/jg2130/jg38691/jg26106/jg23482/jg33679/jg31699/jg4193/novel.455/novel.85/jg2517/jg33261/jg8043/jg4192/jg2948/jg30160/jg23117/novel.490/jg26107/jg26105/jg3461/jg38233/jg4794/jg31098
MF	GO:0004568	chitinase activity	6/960	11/9398	0.000328814	0.013097748	jg34074/jg29753/jg21946/jg12654/jg21947/jg21951
CC	GO:0005618	cell wall	14/214	68/2194	0.004730234	0.212860525	jg1307/jg38108/jg1316/jg3376/jg38109/jg3375/jg20406/jg23674/jg20409/jg20410/jg21321/jg31535/jg3374/jg33498
CC	GO:0030312	external encapsulating structure	14/214	68/2194	0.004730234	0.212860525	jg1307/jg38108/jg1316/jg3376/jg38109/jg3375/jg20406/jg23674/jg20409/jg20410/jg21321/jg31535/jg3374/jg33498
CC	GO:0071944	cell periphery	17/214	110/2194	0.034293692	0.999763843	jg1307/jg12433/jg9686/jg38108/jg30412/jg1316/jg3376/jg38109/jg3375/jg20406/jg23674/jg20409/jg20410/jg21321/jg31535/jg3374/jg33498
