# Supplementary figures and images for: Integration of GWAS and RNA-Seq Analysis to Identify SNPs and Candidate Genes Associated with Alkali Stress Tolerance at the Germination Stage in Mung Bean
Source: Genes (Basel). 2023 Jun 19;14(6):1294. doi: 10.3390/genes14061294 (PMC10298294; doi:10.3390/genes14061294)

**Figure S1.** Estimated population structure of the 277 mungbean accessions with values of  $K = 11$ .

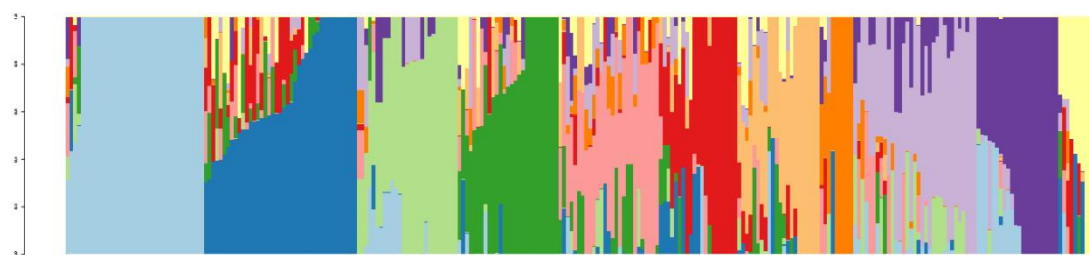

Supplement: Supplementary file 1 [file genes-14-01294-s001.zip › Supplementary Materials/Figure S1. Estimated population structure of the 277 mungbean accessions with values of K = 11.pdf]

**Figure S2.** LD decay analysis of the 11 mungbean groups.

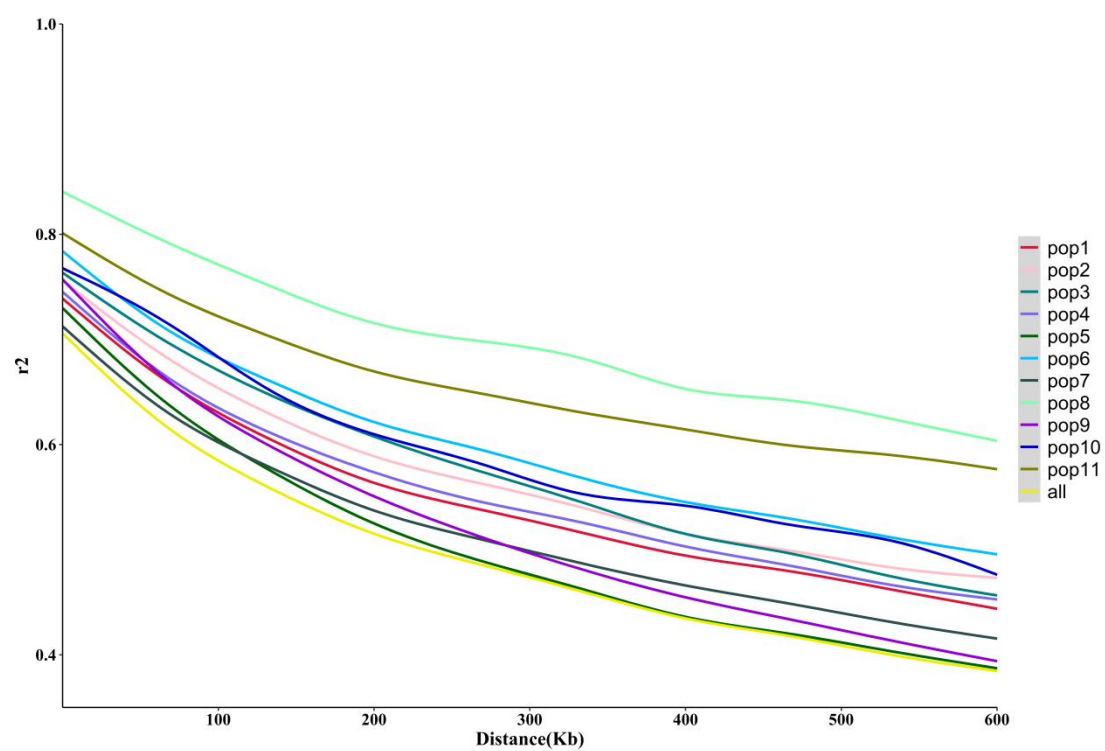

Supplement: Supplementary file 1 [file genes-14-01294-s001.zip › Supplementary Materials/Figure S2. LD decay analysis of the 11 mungbean groups.pdf]

**Figure S3.** Gene Ontology (GO) enrichment analysis of 312 alkali-response genes detected in GWAS.

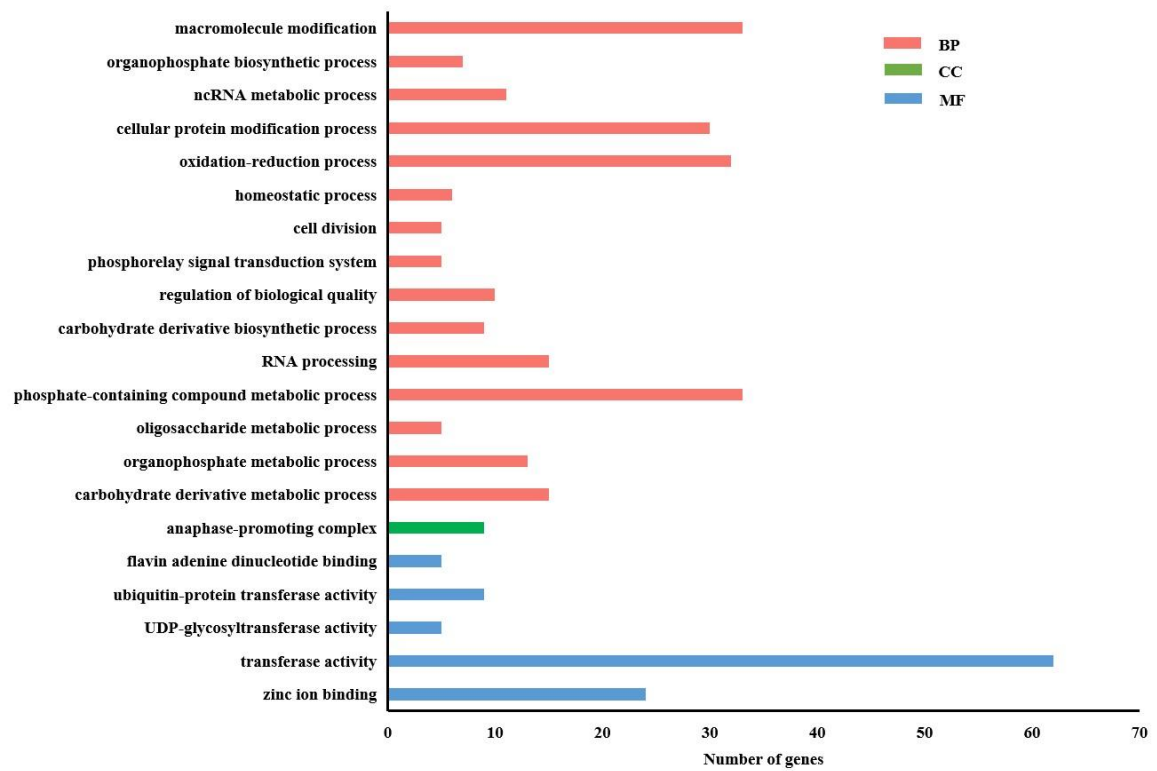

Supplement: Supplementary file 1 [file genes-14-01294-s001.zip › Supplementary Materials/Figure S3. Gene Ontology (GO) enrichment analysis of 312 alkali-response genes detected in GWAS.pdf]

**Figure S4.** Validation of the 14 genes in transcriptome sequencing with qRT-PCR.

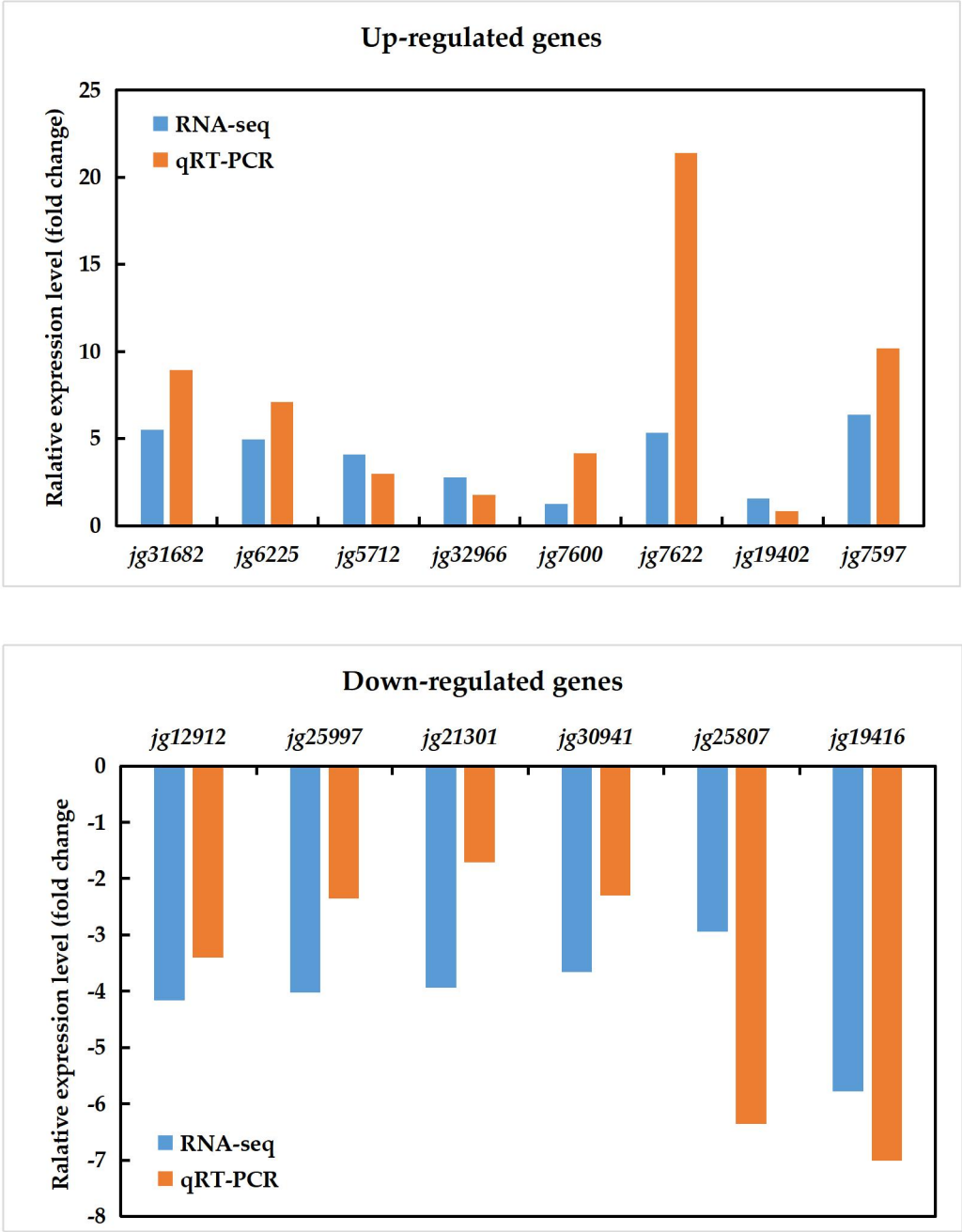

Supplement: Supplementary file 1 [file genes-14-01294-s001.zip › Supplementary Materials/Figure S4. Validation of the 14 genes in transcriptome sequencing with qRT-PCR.pdf]
